# Solvent-Free Mechanochemical Synthesis, Antispasmodic Activity, and Integrated In Silico Mechanistic Analysis of a Dapsone-Derived Phenylaminojuglone

**DOI:** 10.3390/biom16071045

**Published:** 2026-07-17

**Authors:** Ricardo E. Zavaleta-Miñano, Elena Mantilla-Rodríguez, Roberto O. Ybañez-Julca, Daniel Asunción-Alvarez, Cinthya Enriquez-Lara, Justo Huertas-Córdova, Iván M. Quispe-Díaz, Rafael Jara-Aguilar, Edison Vásquez-Corales, Wilfredo O. Gutiérrez-Alvarado, Osvaldo Yañez, Julio Benites

**Affiliations:** 1 Grupo de Investigación en Estudios de Compuestos Naturales y Sintéticos con Actividad a Nivel Sistema Nervioso Central y Musculo Liso, Laboratorio de Farmacología, Facultad de Farmacia y Bioquímica, Universidad Nacional de Trujillo, Trujillo 13011, Peru; rezavaletami@unitru.edu.pe (R.E.Z.-M.); amantilla@unitru.edu.pe (E.M.-R.); hasuncion@unitru.edu.pe (D.A.-A.); iquispe@unitru.edu.pe (I.M.Q.-D.); djara@unitru.edu.pe (R.J.-A.); 2 Programa de Doctorado en Química Medicinal, Facultad de Ciencias de la Salud, Universidad Arturo Prat, Casilla 121, Iquique 1110939, Chile; cenriquez@estudiantesunap.cl (C.E.-L.); jhuertas@estudiantesunap.cl (J.H.-C.); 3 Vicerrectorado de Investigación y Postgrado, Universidad Católica Los Ángeles de Chimbote, Chimbote 02801, Peru; evasquezc@uladech.edu.pe; 4 Facultad de Farmacia y Bioquímica, Universidad Nacional de la Amazonía Peruana, Iquitos 16001, Peru; wilfredo.gutierrez@unapiquitos.edu.pe; 5 Centro de Modelación Ambiental y Dinámica de Sistemas (CEMADIS), Facultad de Ingeniería y Negocios, Universidad de Las Américas, Santiago 7500975, Chile; oyanez@udla.cl; 6 Laboratorio de Química Medicinal, Química y Farmacia, Facultad de Ciencias de la Salud, Universidad Arturo Prat, Casilla 121, Iquique 1110939, Chile

**Keywords:** 1,4-naphthoquinones, mechanochemical synthesis, antispasmodic activity

## Abstract

The structural hybridization of bioactive quinones is a promising strategy for generating pharmacologically active compounds through sustainable synthetic approaches. This study aimed to synthesize and evaluate a dapsone-derived phenylaminojuglone as a potential intestinal smooth muscle relaxant. Juglone (**AJ**) was functionalized with dapsone (**D**) via a solvent-free mechanochemical aza-Michael reaction using silica gel as a mild acid catalyst. The resulting compound (**AJ-D**) was characterized and evaluated in isolated rat ileum preparations. Pharmacological studies were complemented by molecular docking, density functional theory (DFT) calculations, and in silico ADMET predictions. **AJ-D** was obtained with complete regioselectivity at the C-3 position and required shorter reaction times than conventional solution-based methods. The compound exhibited significant spasmolytic and antispasmodic effects under basal conditions and against acetylcholine- and KCl-induced contractions. Its relaxant activity was not significantly affected by muscarinic receptor blockade or K^+^ channel inhibition, whereas verapamil reduced its potency. Calcium reintroduction experiments suggested the involvement of extracellular Ca^2+^ influx pathways. Docking studies suggested favorable interactions with the Ca_V_1.2 L-type calcium channel, whereas DFT and ADMET analyses indicated suitable electronic and drug-like properties. **AJ-D** is a promising juglone-derived scaffold with antispasmodic activity, likely associated with the modulation of extracellular calcium influx pathways involved in intestinal smooth muscle contraction.

## 1. Introduction

Gastrointestinal disorders are frequently associated with abnormal smooth muscle contractility, abdominal pain, and altered intestinal motility [1,2,3]. Therefore, antispasmodic therapy remains an essential component in the symptomatic management of these conditions, particularly through agents capable of modulating muscarinic signaling and/or calcium influx pathways in smooth muscle tissues [4,5,6]. However, currently available therapies may have limitations related to efficacy, adverse effects, or pharmacological selectivity, highlighting the need for new molecular scaffolds with improved smooth muscle relaxant properties [7,8].

Naphthoquinones constitute a privileged class of bioactive compounds, characterized by their rich redox behavior and versatile substitution patterns [9,10]. Both natural and synthetic naphthoquinones exhibit a broad spectrum of biological activities, including anti-inflammatory, antiparasitic, antimicrobial, anticancer, and smooth muscle-modulating effects [11,12,13,14,15,16]. Among these, juglone and several phenylaminojuglone derivatives have demonstrated significant spasmolytic activity in intestinal smooth muscle preparations [17,18].

Previous pharmacological studies suggest that phenylaminojuglones may exert relaxant effects through multiple mechanisms, including the modulation of muscarinic signaling, nitric oxide pathways, potassium channels, and interference with extracellular calcium influx [18]. Inhibition of voltage-dependent calcium entry has emerged as a mechanism associated with a reduction in intestinal contractility. These findings support the use of phenylaminojuglones as promising molecular frameworks for the development of novel smooth muscle relaxants.

Structural hybridization is a useful medicinal chemistry strategy for modulating the physicochemical and pharmacological properties of bioactive scaffolds. Dapsone is a clinically established sulfone-containing drug widely used to treat leprosy and other infectious and inflammatory disorders [19,20]. Beyond its antimicrobial properties, dapsone has been associated with anti-inflammatory and immunomodulatory effects, which have expanded its therapeutic applications in dermatological and immune-mediated conditions [21,22]. In addition, the diphenyl sulfone scaffold provides relevant electronic and physicochemical characteristics, including hydrogen-bonding capacity and modulation of lipophilicity and electronic distribution, making it an attractive pharmacophoric fragment for the development of hybrid molecules.

Therefore, incorporating sulfone-containing aromatic moieties into redox-active quinone systems may yield structurally modified phenylaminojuglones with altered pharmacological and physicochemical properties.

In parallel, sustainable synthetic methodologies have received increasing attention in medicinal chemistry. Mechanochemical and solvent-free approaches reduce solvent consumption, simplify purification procedures, and provide rapid access to functionalized molecules under environmentally friendly conditions [23,24,25,26].

We hypothesized that the structural hybridization of juglone (**AJ**) with dapsone (**D**) would yield a phenylaminojuglone derivative with antispasmodic activity and the ability to modulate the pathways involved in smooth muscle contraction. Furthermore, we anticipated that the electronic features introduced by the sulfone-containing aromatic fragment would influence ligand–target recognition and contribute to the observed pharmacological profile.

In this study, we synthesized a phenylaminojuglone derivative, **AJ-D**, using a solvent-free mechanochemical protocol with silica gel as a solid support under mild conditions. The compound was chemically characterized and evaluated for its relaxant effects on isolated rat ileum preparations under basal conditions and in tissues precontracted with ACh or KCl. Additional pharmacological experiments involving calcium-dependent contraction models and verapamil were performed to explore the possible involvement of extracellular calcium influx in its mechanism of action. Furthermore, molecular docking studies involving the Ca_V_1.2 L-type calcium channel, along with ADMET prediction and density functional theory (DFT) calculations, were performed to obtain complementary mechanistic, electronic, and pharmacokinetic insights into the biological activity of **AJ-D**.

## 2. Materials and Methods

### 2.1. Chemistry

#### 2.1.1. General

All solvents and reagents were purchased from commercial suppliers, including Aldrich (St. Louis, MO, USA) and Merck (Darmstadt, Germany), and were used without further purification. Melting points (mp) were determined using a Stuart Scientific SMP3 apparatus (Staffordshire, UK) and are reported uncorrected. Infrared (IR) spectra were recorded on a Bruker FT-IR spectrophotometer (Vector 22 model; Bruker, Rheinstetten, Germany) using KBr disks. The absorption frequencies are reported in cm^−1^. ^1^H-NMR and ^13^C-NMR spectra were recorded in DMSO-*d*_6_ on a Bruker Avance-400 spectrometer (Bruker, Ettlingen, Germany) at 400 and 100 MHz, respectively. Chemical shifts (δ) are reported in parts per million (ppm) downfield relative to tetramethylsilane (TMS) as the internal standard, and the coupling constants (*J*) are expressed in Hertz (Hz). ^1^H NMR signals are described as follows: s (singlet), br s (broad singlet), d (doublet), and m (multiplet). Two-dimensional NMR (HMBC) was used for signal assignment. Silica gel Merck 60 (70–230 mesh) was used for preparative column chromatography, and silica gel 60F_254_ aluminum plates (Merck) were used for analytical thin-layer chromatography (TLC).

#### 2.1.2. Synthesis of Phenylaminojuglone **AJ-D**

Juglone (**AJ**) was synthesized using a green photochemistry protocol previously developed by our research group based on LED and sunlight irradiation [27]. Phenylaminojuglone **AJ-D** was prepared under solvent-free mechanochemical and solvent-based conditions as follows.

Solvent-free conditions: **AJ** (1 mmol) and dapsone (**D**, 1 mmol) were mixed with silica gel (0.4 g), transferred to a mini-rotary tube shaker, and agitated at 70 rpm at room temperature. The reaction progress was monitored every 10 min by TLC (petroleum ether/ethyl acetate, 3:1 *v*/*v*, R*f* = 0.4) and reached completion after 3 h. Small aliquots of the reaction mixture were suspended in ethyl acetate before TLC analysis. The crude product was purified by silica gel column chromatography (petroleum ether/ethyl acetate) to afford **AJ-D** with a yield of 51.3%.

Solvent-based conditions: **AJ** (1 mmol) and dapsone (**D**, 1 mmol) were dissolved in absolute ethanol (10 mL) and refluxed at 78 °C for 6 h. The reaction progress was monitored by TLC as described above. Purification by silica gel column chromatography (petroleum ether/ethyl acetate) afforded **AJ-D** with a 53% yield.

Both methods afforded a single regioisomer, as confirmed by TLC and ^1^H NMR analyses, with no evidence of regioisomeric byproducts. Compound purity was verified by analytical and preparative TLC, each showing a single spot/band, and further supported by a sharp melting point (300–302 °C). The solvent-free reactions were independently repeated at least three times with reproducible results. The silica gel was readily recovered, washed with acetone, dried at 100 °C, and reused, highlighting the simplicity and sustainability of this protocol.

*3-((4-(4′-aminophenyl)sulfonyl)phenylamino)-5-hydroxy-1,4-naphthoquinone* **AJ-D**. Prepared from juglone (**AJ**) and dapsone (**D**). Compound **AJ-D**, orange solid, mp, 300–302 °C; IR (KBr) ν_max_ cm^−1^: 3462 (OH), 3373 (NH), 1635 (C=O), 1614 (C=O). ^1^H-NMR (400 MHz, DMSO-*d*_6_): δ 6.18 (s, 2H, NH_2_), 6.29 (s, 1H, H–2), 6.64 (d, 2H, *J* = 8.7 Hz, H–3″ + H–5″), 7.27 (d, 1H, *J* = 8.3 Hz, H–6), 7.46 (dd, 1H, *J* = 7.9, 9.5 Hz, H–8), 7.57 (m, 4H, H–3′ + H–5′ + H–2″ + H–6″), 7.73 (dd, 2H, *J* = 7.9, 7.9 Hz, H–7), 7.86 (d, 2H, *J* = 8.6 Hz H–2′ + H–6′), 9.45 (s, 1H, NH), 11.50 (s, 1H, OH). ^13^C-NMR (100 MHz, DMSO-*d*_6_): δ 104.68, 113.09 (2C), 117.74, 122.59 122.89 (2C), 125.63, 127.95 (2C), 128.53, 129.37 (2C), 132.66, 137.56, 138.46, 142.24, 144.94, 153.61, 160.49, 182.41, 185.16. HRMS (APCI): [M + H]^+^ Calcd for C_22_H_16_N_2_O_5_S: 421.08527; found 421.08393 (Figures S1–S5, Supplementary Materials).

### 2.2. In Vitro Experiments

#### 2.2.1. Animals

All procedures complied with the guidelines of the American Veterinary Medical Association (AVMA). The animal study protocol was approved by the Ethics Committee of the Faculty of Pharmacy and Biochemistry, National University of Trujillo (protocol code PR004-2022/CEIFYB; date of approval: 12 December 2022). Male *Rattus norvegicus* Holtzman rats (10–12 weeks old; 170–200 g) were housed at 22–25 °C under a 12 h light/dark cycle with free access to standard chow (Molinorte S.A.C., Trujillo, Peru) and water ad libitum.

#### 2.2.2. Preparation of Rat Ileum

The animals were sacrificed by cervical dislocation. A 2.5 cm ileal segment, located at least 10 cm proximal to the ileocecal valve, was excised and placed in a Petri dish containing Tyrode’s solution with the following composition (in mM): NaCl 136.9, KCl 2.68, CaCl_2_ 1.8, MgCl_2_ 1.05, NaHCO_3_ 11.9, NaH_2_PO_4_ 0.42, and *D*-glucose 5.55 [28]. The ileum was selected over the duodenum and jejunum because of its lower stiffness and tension, along with greater wall thickness and luminal area, which make it especially suitable for mechanical studies [29,30]. The ileum samples were placed in an isolated organ chamber (Automatic Organ Bath; PanLab-Harvard Apparatus, Barcelona, Spain) containing 25 mL Tyrode’s solution. The chamber was maintained at 37 °C (LE 13206 Thermostat; Panlab Harvard Apparatus, Barcelona, Spain) and continuously aerated with a mixture of 95% O_2_ and 5% CO_2_ (pH 7.4). The resting tension was set at 1 g. The ileum sections were attached to the bottom of a stainless steel hook and secured at the top with a black braided silk thread (6-0/TC-15, RS No. DM0441N, Cirugia Peruana, Lima, Peru) to an isometric transducer (Force Transducer 0–50 g, MLTF050/ST; AD Instruments Pty Ltd., New South Wales, Australia). Isometric transducers were used to obtain precise and reproducible measurements of agonist-induced contractile force under constant muscle length, thereby minimizing mechanical artifacts and allowing reliable pharmacological concentration–response analysis [31,32]. The transducer was connected to a PowerLab 26T data acquisition system (ADInstruments, Pty Ltd., New South Wales, Australia) for continuous monitoring of intestinal reactivity using LabChart 8 software (ADInstruments Pty Ltd., New South Wales, Australia).

#### 2.2.3. Effect of Phenylaminojuglone **AJ-D** on the Basal Tone of Rat Ileum

The contractility of the rat ileum was evaluated in response to phenylaminojuglone **AJ-D** through separate cumulative concentration–response experiments. A series of different concentrations of **AJ-D** (10^−6^, 3.16 × 10^−6^, 10^−5^, 2.5 × 10^−5^, 5 × 10^−5^, 7.5 × 10^−5^, 10^−4^, 2.5 × 10^−4^, 5 × 10^−4^ M) were administered at intervals of 3 min.

#### 2.2.4. Spasmolytic Effect of Phenylaminojuglone **AJ-D** on ACh and KCl Pre-Contracted Rat Ileum

Neurotropic spasm was induced using ACh, and musculotropic spasm was induced using a high-KCl-containing solution. Isolated ileal sections were treated with ACh (10^−5^ M) or KCl (60 mM) for 10 min or until a stable contractile plateau (plateau) was reached. Then **AJ-D** (10^−6^, 3.16 × 10^−6^, 10^−5^, 2.5 × 10^−5^, 5 × 10^−5^, 7.5 × 10^−5^, 10^−4^, 2.5 × 10^−4^, 5 × 10^−4^ M) concentrations were sequentially added to the tissue chamber. Relaxation was expressed as a percentage of the initial contractile response induced by ACh or KCl. Values greater than 100% indicate relaxation below the initial basal tone after complete reversal of the induced contraction.

#### 2.2.5. Effect of Phenylaminojuglone **AJ-D** on ACh-Induced Dose–Response Curves in Rat Ileum

The effect of **AJ-D** on contractility in response to ACh was assessed using concentration–response experiments (ACh, 10^−10^ to 10^−4^ M) before and after **AJ-D** (10^−10^ to 10^−4^ M) administration in the same experiment. Experiments similar to those described above but contracting the ileal sections with KCl (1–60 mM) were performed.

#### 2.2.6. Evaluation of the Possible Involvement of Muscarinic Receptors in **AJ-D**-Induced Relaxation

To investigate the role of **AJ-D** in muscarinic receptor activity, experiments were carried out in the absence or presence of atropine (1 µM), a non-selective muscarinic receptor antagonist. First, the tissue was stabilized for 1 h and then washed every 15 min (4–5 times) with Tyrode’s solution. The tissue was then pre-incubated for 20 min with 1 µM atropine, followed by successive additions of different concentrations of **AJ-D** (10^−6^–10^−3^ M, respectively).

#### 2.2.7. Effect of Phenylaminojuglone **AJ-D** on the Activity of Potassium Channels

The role of K^+^ channels in **AJ-D**-induced relaxation was investigated by pre-incubating ileum rat sections for 20 min with four K^+^ channel blockers: 10 μM glibenclamide (an ATP-sensitive K^+^ channel blocker), 10 μM barium chloride (an inward rectifier K^+^ channel blocker), 1 mM 4-aminopyridine (4-AP, a voltage-gated K^+^ channel blocker), and 1 mM tetraethylammonium (TEA, a non-selective Ca^2+^-activated K^+^ channel blocker). Tissue sections were stabilized for 1 h and washed every 15 min (4–5 times). The tension was readjusted to 1 g if necessary. The tissue was then pre-incubated with any of the K^+^ channel blockers, followed by serial addition of **AJ-D** (10^−6^, 10^−5.5^, 10^−5^, 10^−4.5^, 10^−4^, 10^−3.5^, 10^−3^ M) at 3 min intervals for each successive concentration, and the response was recorded.

#### 2.2.8. Evaluation of the Role of Extracellular Calcium in **AJ-D**-Induced Relaxation

To investigate the effect of extracellular calcium on ileal contraction induced by high external K^+^ concentrations, we followed a previously described protocol with slight modifications [17]. In addition to Tyrode’s solution, a Ca^2+^-free solution was prepared with the following composition (in mM): KCl 50, NaCl 91.04, MgCl_2_ 1.05, NaHCO_3_ 11.87, NaH_2_PO_4_ 0.41, and D-glucose 5.55. Initially, the tissue was stabilized in normal Tyrode’s solution before being replaced with Ca^2+^-free Tyrode’s solution (no calcium added plus 0.1 mM EDTA). Then, 10 min after the addition of the calcium-free solution, the intestinal segments were contracted with 60 mM KCl, followed by the cumulative addition of CaCl_2_ at increasing concentrations (0.1, 0.3, 0.6, 1.0, 2.0, and 5.0 mM). Subsequently, the tissue was washed 2–3 times with normal Tyrode’s solution.

After this step, the tissue was washed with Ca^2+^-free Tyrode’s solution and pre-incubated with **AJ-D** for 5 min. Following pre-incubation, tissue sections were contracted with 60 mM KCl, and increasing concentrations of CaCl_2_ (0.1, 0.3, 0.6, 1.0, 2.0, and 5.0 mM) were cumulatively added.

To evaluate whether the dependence on extracellular Ca^2+^ involved voltage-gated calcium channels, experiments were performed in the presence and absence of verapamil, a voltage-gated calcium channel blocker [33]. Initially, the tissue sections were stabilized for 1 h and then washed at 15 min intervals (4–5 times). The tension was readjusted to 1 g if necessary. Tissue sections were pre-incubated with verapamil (10^−5^ M) for 20 min. **AJ-D** was then added at concentrations of 10^−6^, 10^−5.5^, 10^−5^, 10^−4.5^, 10^−4^, 10^−3.5^, 10^−3^ M at 3 min intervals.

### 2.3. In Silico Studies

#### 2.3.1. Molecular Docking

Phenylaminojuglone **AJ-D** was docked into the L-type voltage-gated calcium channel Cav1.2 (PDB ID: 8WE8) [34], a molecular target associated with smooth muscle contractility and extracellular calcium influx.

Potential binding cavities were identified using the CurPocket algorithm, and docking grids were defined around the most relevant pockets based on the cavity geometry and residue composition. For the Cav1.2 channel, docking calculations were performed in the transmembrane cavity (CurPocket C2) associated with calcium channel modulation.

The molecular docking process utilized AutoDock (version 4.2.1), AutoDock Vina (version 1.0.2) [35], and AutoDockTools packages [36]. Docking parameters and protocols were established following the methodology previously reported by Ybañez-Julca et al. [17] with minor modifications. Docking protocols were validated by re-docking the co-crystallized ligands into their respective binding sites. The obtained poses reproduced the experimental orientations with RMSD values below 2.0 Å.

#### 2.3.2. ADMET and Extended In Silico Toxicity Predictions

The molecular structure of **AJ-D** was represented in SMILES format and submitted to the pkCSM online platform (http://biosig.unimelb.edu.au/pkcsm/prediction, accessed on 31 December 2025) [37] to predict absorption, distribution, metabolism, excretion, and toxicity (ADMET) properties. In addition, an extended in silico toxicity assessment was performed using the ProTox-3.0 webserver (https://tox.charite.de/protox3/, accessed on 4 May 2026) [38]. The ProTox-3.0 predictions included acute oral toxicity (LD_50_ and toxicity class), organ toxicity endpoints, and additional toxicity endpoints, including carcinogenicity, immunotoxicity, mutagenicity, and cytotoxicity. The prediction accuracy and average structural similarity values provided by the platform were also considered during the interpretation of the results.

#### 2.3.3. Density Functional Theory (DFT) Calculation

The three-dimensional molecular structures of **AJ-D** and verapamil were constructed using Discovery Studio Visualizer v21.0.1, ensuring consistent and reliable representation of the compounds for subsequent reactivity analyses. Starting from these initial geometries, the potential energy surface was explored to identify the most stable conformer of each molecule using the CREST v3.0.2 (Conformer–Rotamer Ensemble Sampling Tool) program [39], an open-source code designed for automated exploration of molecular conformational space, in combination with the GFN2-xTB method [40].

The lowest-energy conformer obtained for each system was selected for full geometry optimization at the M06-2X/6-311++G(d,p) [41,42] level of theory using the Gaussian16 software package [43]. Vibrational frequency calculations were performed at the same level to verify that the optimized structures corresponded to true minima on their respective potential energy surfaces (i.e., absence of imaginary frequencies). Based on these DFT results, a set of global and local reactivity descriptors was computed, as summarized in Table 1.

The evaluated descriptors included the HOMO–LUMO gap, which reflects the energy difference between the highest occupied molecular orbital (HOMO) and the lowest unoccupied molecular orbital (LUMO) and is closely related to molecular stability and electronic excitations. Additionally, the ionization potential (*IP*), electron affinity (*EA*), electronegativity (*χ*), global hardness (*η*), electrophilicity index (*ω*), electrodonating (*ω*^−^), electroaccepting (*ω*^+^), and net electrophilicity (Δ*ω*^±^) indices were determined to characterize different aspects of the electronic structure and reactivity of **AJ-D** and verapamil. Furthermore, Fukui functions (*f*^+^, *f*^−^, and *f*^0^) were calculated to identify nucleophilic and electrophilic sites within the molecules, providing a detailed picture of the local reactivity patterns.

Furthermore, three-dimensional molecular electrostatic potential (MEP) surfaces were generated to obtain a more comprehensive picture of the electronic structures of **AJ-D** and verapamil. These color-coded maps depict the spatial distribution of the electrostatic potential, with positive electrostatic potential regions (blue) indicating electron-deficient sites that are more susceptible to nucleophilic interactions, whereas negative potential regions (green to red) correspond to electron-rich areas that may preferentially interact with electrophilic or positively charged species [52]. This analysis provides key insights into the reactivity patterns and site-specific noncovalent interactions of the studied molecules.

### 2.4. Statistical Analysis

Statistical analyses were performed using GraphPad Prism (version 8.0.2; GraphPad Software, San Diego, CA, USA). Dose–response curves were compared using non-linear regression analysis with a three-parameter Hill function. Differences between groups were evaluated using two-way ANOVA, followed by Bonferroni’s post hoc test. For comparisons between the two groups, either a paired or unpaired Student’s *t*-test was applied, as appropriate. Data normality was assessed using the Shapiro–Wilk test.

## 3. Results

### 3.1. Solvent-Free Synthesis of Phenylaminojuglone ***AJ-D***

The reaction yields and reaction times obtained for **AJ-D** under solvent-free conditions and in ethanol at room temperature are summarized in Table 2. Under solvent-free conditions, the amination of juglone with dapsone proceeded efficiently, reaching completion within 3 h and affording the corresponding phenylaminojuglone **AJ-D** in moderate yield. In contrast, when the reaction was performed in ethanol, a significantly longer reaction time was required for completion.

After completion of the reaction, the spent silica gel was readily recovered by simple filtration, washed with acetone, and dried at 100 °C prior to reuse, highlighting the operational simplicity and sustainability of the solvent-free protocol. The reaction afforded a single regioisomer, as confirmed by TLC and ^1^H-NMR analyses, with no evidence of additional regioisomeric products.

The exclusive formation of C-3-substituted phenylaminojuglone indicates that the reaction proceeded with high regioselectivity under the applied mechanochemical conditions. The molecular structure of **AJ-D** was confirmed by IR, 1D and 2D NMR, and HRMS analyses (Figures S1–S5, Supplementary Materials). Key HMBC correlations established the connectivity between the juglone and dapsone moieties. The NH proton of the dapsone amino group (δ_H_ 9.45 ppm) showed a three-bond correlation with the juglone carbonyl carbon C-4 (δ_C_ 185.16 ppm), confirming the attachment of the amino substituent at C-3 of the juglone scaffold. Likewise, the quinonic H-2 proton (δ_H_ 6.29 ppm) correlated with C-4, while the H-8 proton (δ_H_ 7.46 ppm) exhibited an HMBC cross-peak with the C-1 carbonyl carbon (δ_C_ 182.41 ppm), corroborating the regiochemistry of the 1,4-naphthoquinone nucleus (Figure S4, Supplementary Materials). The ^13^C NMR spectrum further supported the proposed structure by displaying characteristic quinone carbonyl signals at δ_C_ 182.41 (C-1) and 185.16 ppm (C-4).

### 3.2. Spasmolytic Activity

#### Effect of Phenylaminojuglone **AJ-D** on Rat Ileum Basal Tone

**Figure 1:**
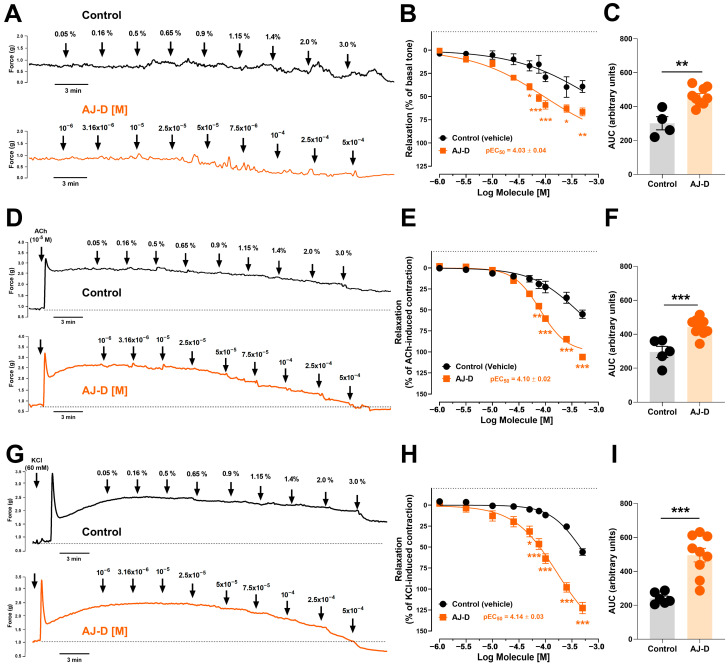
Relaxant effects of phenylaminojuglone **AJ-D** on rat ileal smooth muscle under basal and pre-contracted conditions. Representative recordings showing the effects of cumulative concentrations of **AJ-D** on spontaneous ileal contractions (**A**), ileal segments pre-contracted with ACh (10^−5^ M) (**D**), or KCl (60 mM) (**G**). The corresponding concentration–response curves are shown in panels (**B**), (**E**), and (**H**), respectively, and are expressed as the percentage of relaxation relative to the basal tone, ACh-induced contraction, or KCl-induced contraction. Panels (**C**,**F**,**I**) show the area under the concentration–response curve (AUC), used to quantify the overall relaxant effect. Data are presented as mean ± SEM (*n* = 4–9 ileal segments). Concentration–response curves were fitted using nonlinear regression (Hill equation). Statistical analysis was performed using two-way ANOVA followed by Bonferroni’s post hoc test for concentration–response curves and unpaired Student’s *t*-test for AUC comparisons. * *p* < 0.05, ** *p* < 0.01, *** *p* < 0.001 vs. control. Values > 100% indicate relaxation below basal tone.

The spasmolytic effect of **AJ-D** was evaluated using isolated rat ileum preparations that exhibited spontaneous contractile activity. As shown in Figure 1A, the cumulative addition of **AJ-D** resulted in a concentration-dependent reduction in the basal ileal tone. The concentration–response curve (Figure 1B) demonstrated a progressive relaxation of the spontaneous contractile activity. In particular, 5 × 10^−5^ M **AJ-D** produced a significant relaxation of 39.4 ± 3.3% (*p* < 0.05). At the highest concentration tested (5 × 10^−4^ M), **AJ-D** induced marked relaxation of the ileal smooth muscle, reaching 66.4 ± 4.1% (*p* < 0.01 vs. control group). Moreover, **AJ-D** exhibited a lower EC_50_ value (9.45 × 10^−5^ M) compared to the control group (7.67 × 10^−4^ M), indicating greater spasmolytic potency.

To further evaluate the overall relaxant effect, the area under the concentration–response curve (AUC) was calculated (Figure 1C). Under these conditions, **AJ-D** exhibited a significantly higher AUC value (460 ± 17 arbitrary units, a.u.) than that of the control group (301 ± 39 a.u.; *p* < 0.01). These findings indicate that **AJ-D** exerts a significant relaxant effect on spontaneous rat ileal contractions in vitro.

### 3.3. Antispasmodic Activity of Phenylaminojuglone ***AJ-D*** on the Rat Ileum

#### 3.3.1. Effect of Phenylaminojuglone **AJ-D** on Intestinal Sections Pre-Contracted with ACh

To evaluate the effects of **AJ-D** on receptor-mediated contraction, the ileal segments were pre-contracted with ACh, 10^−5^ M. As shown in Figure 1D, the cumulative addition of **AJ-D** progressively reduced the sustained contractile tone induced by ACh. The concentration–response curve shown in Figure 1E demonstrates the concentration-dependent relaxation of ACh-induced contraction.

At a concentration of 7 × 10^−5^ M, **AJ-D** induced significant relaxation (45.4 ± 3.4%; *p* < 0.001) compared with that in vehicle-treated tissues (19.0 ± 5.3%). At the highest concentration tested (5 × 10^−4^ M), **AJ-D** markedly reduced ACh-induced contraction, producing 106.1 ± 2.4% relaxation (*p* < 0.001, compared to control). Furthermore, **AJ-D** exhibited a lower EC_50_ value (7.97 × 10^−5^ M) compared to the vehicle control group (4.40 × 10^−4^ M), indicating greater inhibitory potency against ACh-induced pharmacomechanical contraction.

The overall relaxant effect was further evaluated using AUC analysis (Figure 1F). Vehicle-treated tissues exhibited an AUC value of 296 ± 33 a.u., whereas **AJ-D** significantly increased cumulative relaxation (442 ± 17 a.u.; *p* < 0.01 vs. control group). These findings indicate that **AJ-D** significantly inhibits ACh-induced pharmacomechanical contractions in the rat ileum.

#### 3.3.2. Effect of Phenylaminojuglone **AJ-D** on KCl-Precontracted Intestinal Sections

As shown in Figure 1G, the cumulative addition of **AJ-D** induced relaxation in the ileal segments that were previously contracted with KCl (60 mM). The concentration–response curve presented in Figure 1H demonstrates the concentration-dependent relaxation of KCl-induced contractions.

Starting at 7.5 × 10^−5^ M, **AJ-D** significantly reduced the contractile tone (46.3 ± 6.4%; *p* < 0.001 vs. control group). At the highest concentration tested (5 × 10^−4^ M), **AJ-D** induced marked relaxation, reaching 122.6 ± 6.6% (*p* < 0.001, compared to control). In addition, **AJ-D** exhibited a lower EC_50_ value (7.25 × 10^−5^ M) compared with the vehicle control group (4.49 × 10^−4^ M), indicating greater potency in inhibiting KCl-induced contraction.

The overall relaxant effect was further assessed by calculating the area under the concentration–response curve (Figure 1I). Vehicle-treated tissues exhibited an AUC value of 241 ± 14 a.u., whereas **AJ-D** significantly increased cumulative relaxation (495 ± 40 a.u.; *p* < 0.001 vs. control). These findings indicate that **AJ-D** exerts significant relaxant effects on KCl-induced tonic contractions in the rat ileum.

### 3.4. Antispasmodic Activity of Phenylaminojuglone ***AJ-D***

#### 3.4.1. Effect of Phenylaminojuglone **AJ-D** on ACh-Induced Intestinal Contractility

**Figure 2:**
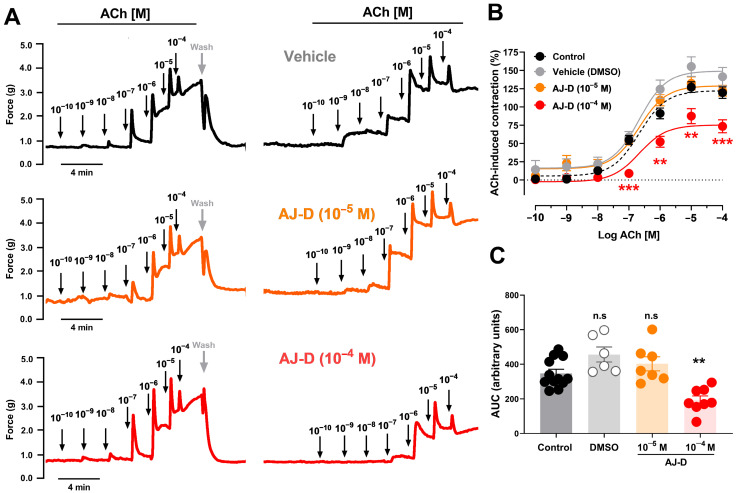
Antispasmodic effects of phenylaminojuglone **AJ-D** on rat ileal smooth muscle. (**A**) Representative traces showing the cumulative concentration–response curves to ACh (10^−10^ to 10^−4^) in isolated rat ileal segments under control conditions, in the presence of vehicle (DMSO), and after 20 min preincubation with **AJ-D** (10^−5^ or 10^−4^ M). (**B**) Concentration–response curves for ACh-induced contractions in control, vehicle-treated, and **AJ-D** preincubated tissues. (**C**) Area under the concentration–response curve (AUC) used to quantify the overall contractile response to ACh in the presence or absence of **AJ-D**. Data are expressed as mean ± SEM (*n* = 6–12 ileal segments). The colored lines represent the nonlinear regression fits using the Hill equation. Statistical analysis was performed using two-way ANOVA for dose–response curves and one-way ANOVA for AUC, followed by Bonferroni’s post hoc test. Significance: ** *p* < 0.01; *** *p* < 0.001 vs. control; n.s. = not significant.

To further investigate the mechanism underlying the antispasmodic activity of **AJ-D**, additional experiments were performed to evaluate its effect on ACh-induced contractions in the isolated rat ileum. Pre-incubation with the vehicle (DMSO) did not significantly alter the contractile response to ACh. As shown in Figure 2A, the cumulative addition of ACh (10^−10^–10^−4^ M) produced a typical concentration-dependent increase in the contractile force of the ileum under control conditions. Preincubation with **AJ-D** (10^−4^ M) for 20 min markedly attenuated the contractile responses induced by increasing concentrations of ACh. In contrast, 10^−5^ M **AJ-D** produced only a slight tendency toward inhibition without reaching statistical significance.

At an ACh concentration of 10^−7^ M, the control tissues generated contractions corresponding to 54.8 ± 6.0% of the maximal response. This response was not significantly altered by 10^−5^ M **AJ-D** (53.1 ± 5.0%; *p* > 0.05, Figure 2B). However, preincubation with 10^−4^ M **AJ-D** significantly reduced the contraction induced by ACh to 9.0 ± 2.3% (*p* < 0.001 vs. control). Similarly, the maximal contractile response elicited by 10^−4^ M ACh (119.5 ± 7.9% in control tissues) was significantly attenuated by **AJ-D** at 10^−4^ M (73.4 ± 9.0%; *p* < 0.001), whereas **AJ-D** at 10^−5^ M did not significantly modify the maximal response (122.1 ± 7.4%; *p* > 0.05).

Analysis of agonist potency revealed that preincubation with **AJ-D** (10^−4^ M) significantly reduced the pEC_50_ value of ACh-induced contractions compared to that in control tissues (6.23 ± 0.16 vs. 6.83 ± 0.10; *p* < 0.01), indicating a rightward shift of the concentration–response curve. In contrast, neither the vehicle (DMSO) nor 10^−5^ M **AJ-D** significantly altered pEC_50_ values.

Consistent with these findings, the analysis of the area under the concentration–response curve (AUC) demonstrated that tissues preincubated with **AJ-D** (10^−4^ M) exhibited a significant reduction in cumulative contractile activity (193 ± 25 a.u.) compared to control tissues (348 ± 24 a.u.; *p* < 0.01) (Figure 2C). No significant differences were observed for **AJ-D** at 10^−5^ M. These results indicate that **AJ-D** partially inhibits ACh-induced contractions in the rat ileum, reducing both the maximal contractile response and agonist potency at higher **AJ-D** concentrations.

#### 3.4.2. Influence of Phenylaminojuglone **AJ-D** on KCl-Induced Intestinal Contractility

**Figure 3:**
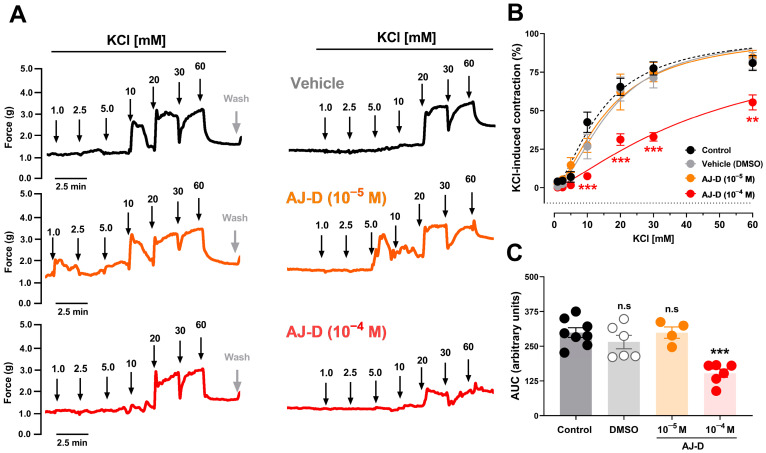
Antispasmodic effects of phenylaminojuglone **AJ-D** on rat ileal smooth muscle pre-contracted with KCl. (**A**) Representative traces showing the cumulative concentration–response curves to KCl (1–60 mM) in isolated rat ileal segments under control conditions, in the presence of vehicle (DMSO), and after 20 min preincubation with **AJ-D** (10^−5^ or 10^−4^ M). (**B**) Concentration–response curves for KCl-induced contractions in control, vehicle-treated, and **AJ-D** preincubated tissues. (**C**) Area under the concentration–response curve (AUC) used to quantify the overall contractile response to KCl in the presence or absence of **AJ-D**. Data are expressed as mean ± SEM (*n* = 4–8 ileal segments). The colored lines represent the nonlinear regression fits using the Hill equation. Statistical analysis was performed using two-way ANOVA for dose–response curves and one-way ANOVA for AUC, followed by Bonferroni’s post hoc test. Significance: ** *p* < 0.01; *** *p* < 0.001 vs. control; n.s. = not significant.

To further explore the mechanism underlying the spasmolytic activity of **AJ-D**, concentration–response curves for KCl (1–60 mM) were generated using isolated rat ileal preparations. As shown in Figure 3A, the cumulative addition of KCl produced a typical concentration-dependent increase in the contractile force under control conditions. Preincubation with the vehicle (DMSO) did not significantly alter the contractile response to KCl treatment. Similarly, 10^−5^ M **AJ-D** produced only a slight tendency toward inhibition and did not significantly reduce KCl-induced contractions. In contrast, tissues preincubated for 20 min with **AJ-D** at 10^−4^ M exhibited a marked attenuation of contractile responses induced by increasing concentrations of KCl.

For instance, contractions induced by 10 mM KCl (control: 42.4 ± 6.6%) were not significantly affected by 10^−5^ M **AJ-D** (27.4 ± 4.5%; *p* > 0.05), whereas a significant reduction was observed in tissues treated with 10^−4^ M **AJ-D** (7.4 ± 1.9%; *p* < 0.001) (Figure 3B). Similarly, contractions induced by 60 mM KCl (control: 80.9 ± 5.9%) were significantly reduced by 10^−4^ M **AJ-D** (55.4 ± 4.9%; *p* < 0.05), whereas no significant effect was observed with 10^−5^ M **AJ-D** (84.6 ± 4.5%; *p* > 0.05).

Analysis of agonist potency revealed that preincubation with **AJ-D** (10^−4^ M) significantly increased the EC_50_ value of KCl-induced contractions compared to control tissues (48.4 ± 3.3 mM vs. 14.2 ± 1.0 mM; *p* < 0.001), indicating a rightward displacement of the concentration–response curve. In contrast, the EC_50_ values obtained in the presence of the vehicle (DMSO) or 10^−5^ M **AJ-D** were not significantly different from those of the control tissues.

Consistent with these findings, analysis of the area under the concentration–response curve (AUC) demonstrated that tissues preincubated with **AJ-D** (10^−4^ M) exhibited a significant reduction in the overall contractile response (153 ± 15 a.u.) compared to control tissues (299 ± 17 a.u.; *p* < 0.001) (Figure 3C). No significant differences were observed for **AJ-D** at 10^−5^ M. Collectively, these results suggest that **AJ-D** attenuates KCl-induced electromechanical contractions in the rat ileum, with significant inhibitory effects observed predominantly at higher concentrations.

### 3.5. Mechanisms of Action Underlying the Effect of Phenylaminojuglone ***AJ-D*** on Smooth Muscle Function

#### 3.5.1. Effect of Phenylaminojuglone **AJ-D** on Muscarinic Receptors

Following the observation that **AJ-D** reduced ACh-induced ileal contractions, additional experiments were performed to determine whether its relaxant effect involved the modulation of muscarinic receptors. For this purpose, ileal segments were preincubated with atropine (1 µM), a non-selective muscarinic receptor antagonist (M_1_–M_5_), prior to **AJ-D** administration.

As shown in Figure S6A (Supplementary Materials), pretreatment with atropine (1 µM) for 20 min did not significantly modify the relaxant effect of **AJ-D** on the basal ileal tone. The relaxation produced by **AJ-D** in the control tissues (58.9 ± 4.4%) was not significantly different from that observed in the atropine-pretreated tissues (65.7 ± 6.2%; *p* > 0.05).

Consistent with these findings, the AUC analysis revealed no significant differences between the control tissues (301 ± 17 a.u.) and atropine-pretreated tissues (359 ± 33 a.u.; *p* > 0.05) (Figure S6B, Supplementary Materials). Taken together, these results indicate that muscarinic receptor blockade does not significantly alter the relaxant effect of **AJ-D**, suggesting that its spasmolytic activity is unlikely to involve the antagonism of muscarinic receptors.

#### 3.5.2. Effect of Four K^+^ Channel Blockers on the **AJ-D**-Induced Relaxation in Rat Ileum

To investigate whether K^+^ channels contribute to the relaxant effect of **AJ-D**, ileal segments were preincubated with different K^+^ channel blockers prior to **AJ-D** treatment. The blockers used were BaCl_2_ (10 µM; inward rectifier K^+^ channel blocker, K_IR_), glibenclamide (10 µM; ATP-sensitive K^+^ channel blocker, K_ATP_), 4-aminopyridine (4-AP, 1 mM; voltage-gated K^+^ channel blocker, K_V_), and tetraethylammonium (TEA, 1 mM; Ca^2+^-activated K^+^ channel blocker, K_Ca_) (Figure S7, Supplementary Materials).

Under control conditions, **AJ-D** induced relaxation of 66.4 ± 4.1%, with an AUC value of 301.2 ± 17.3 a.u. Blockade of K_IR_ channels with BaCl_2_ did not significantly modify the relaxant response induced by **AJ-D** (74.9 ± 6.7%; *p* > 0.05), and the AUC values remained unchanged (323 ± 28 a.u.; *p* > 0.05).

Similarly, inhibition of K_ATP_ channels with glibenclamide did not alter **AJ-D**-induced relaxation (70.7 ± 6.3%; *p* > 0.05), with no significant differences in the AUC values (286 ± 28 a.u.; *p* > 0.05). Likewise, pretreatment with 4-AP or TEA did not significantly affect **AJ-D**-induced relaxation or the corresponding AUC values.

Taken together, these findings indicate that blockade of K_IR_, K_ATP_, K_V_, or K_Ca_ channels does not significantly alter the relaxant effect of **AJ-D**, suggesting that activation of K^+^ channels is unlikely to be the major mechanism underlying **AJ-D**-induced relaxation in rat ileal smooth muscle.

#### 3.5.3. Extracellular Ca^2+^ Dependence of Phenylaminojuglone **AJ-D** Effect

To further investigate the mechanism underlying the spasmolytic activity of **AJ-D**, we examined the possible involvement of extracellular Ca^2+^ influx.

**Figure 4:**
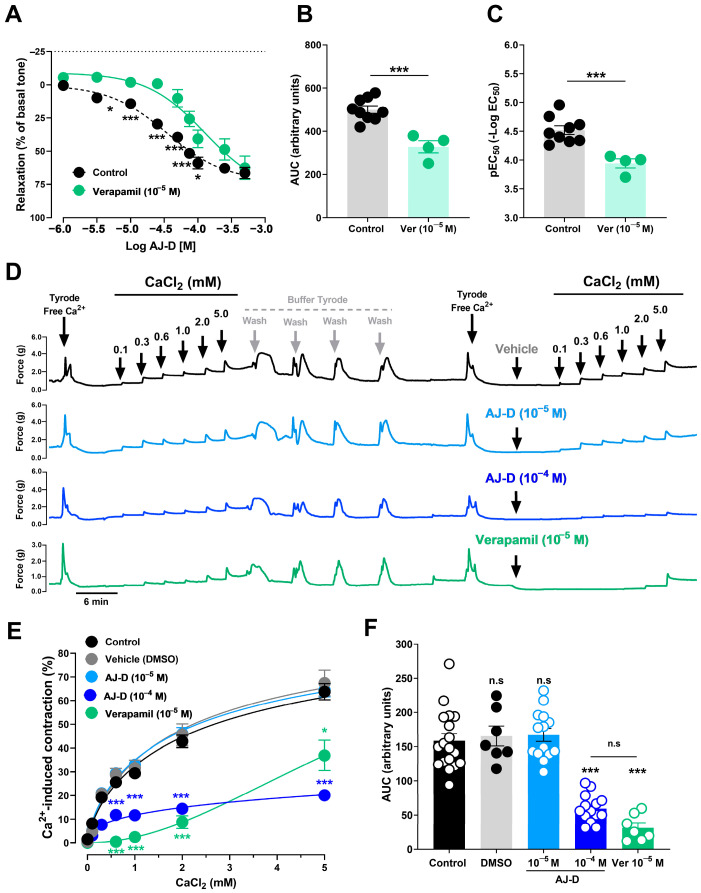
Role of voltage-dependent L-type Ca^2+^ channels (LTCCs) in the antispasmodic effect of phenylaminojuglone **AJ-D** on rat ileal smooth muscle. (**A**) Percentage of relaxation. (**B**) Area under the concentration–response curve (AUC). (**C**) Potency expressed as pEC_50_ values illustrating the relaxant effect of **AJ-D** in the presence and absence of verapamil (10^−5^ M). (**D**) Representative traces showing cumulative CaCl_2_-induced contractions (0.1–5.0 mM) in rat ileum precontracted with KCl (60 mM) and maintained in Ca^2+^-free Tyrode’s solution containing 0.1 mM EDTA under control conditions and after treatment with vehicle (DMSO), **AJ-D** (10^−5^ or 10^−4^ M), or verapamil (10^−5^ M). (**E**) Concentration–response curves for CaCl_2_-induced contractions in control tissues and corresponding treatment groups. (**F**) Corresponding AUC analysis of CaCl_2_-induced contractile responses. In the concentration–response curves, each point represents the mean of the maximal response as a percentage ± SEM (*n* = 4–18 ileal segments). Concentration–response curves were analyzed using two-way ANOVA followed by Bonferroni’s post hoc test, whereas AUC and pEC_50_ values were analyzed using one-way ANOVA or an unpaired Student’s *t*-test, as appropriate. Significance: * *p* < 0.05; *** *p* < 0.001 vs. control; n.s. = not significant.

Initially, the relaxant effect of **AJ-D** on the basal ileal tone was evaluated in the presence of verapamil (10^−5^ M), a selective L-type Ca^2+^ channel blocker. As shown in Figure 4A, preincubation with verapamil significantly reduced the relaxation induced by 5 × 10^−5^ M **AJ-D** (absence of verapamil: 39.4 ± 3.3% vs. presence of verapamil: 10.2 ± 6.5%; *p* < 0.001).

Consistent with this observation, AUC analysis demonstrated that verapamil significantly attenuated the overall relaxant response produced by **AJ-D** (control: 500 ± 17 a.u. vs. verapamil: 328 ± 28 a.u.; *p* < 0.001) (Figure 4B).

At higher **AJ-D** concentrations (2.5 × 10^−4^ and 5 × 10^−4^ M), the relaxant responses in the presence of verapamil were comparable to those observed under control conditions. Similarly, verapamil significantly reduced the pEC_50_ value of **AJ-D** (control: 4.52 ± 0.08 vs. verapamil: 3.94 ± 0.08; *p* < 0.001; Figure 4C), indicating a rightward shift of the concentration–response curve, whereas Emax remained largely unchanged.

To further investigate the role of extracellular Ca^2+^ influx, cumulative CaCl_2_-induced contractions were evaluated in KCl-depolarized ileal tissues maintained in Ca^2+^-free Tyrode’s solution containing 0.1 mM EDTA. Under these conditions, the addition of extracellular Ca^2+^ produced a concentration-dependent increase in the contractile force (Figure 4D).

Pre-incubation with **AJ-D** (10^−5^ M) did not significantly alter the CaCl_2_-induced contractions. In contrast, 10^−4^ M **AJ-D** markedly reduced Ca^2+^-induced contractile responses, producing an effect comparable to that of verapamil (Figure 4E).

These findings were further supported by AUC analysis (Figure 4F), which demonstrated a significant reduction in cumulative CaCl_2_-induced contractility following treatment with **AJ-D** at 10^−4^ M.

Taken together, these results suggest that the relaxant effect of **AJ-D** is associated with a reduction in extracellular Ca^2+^ influx, supporting the possible contribution of voltage-dependent calcium entry pathways to its spasmolytic activity.

To facilitate a quantitative comparison of the effects of **AJ-D** across different experimental conditions, pharmacological parameters, including potency (pEC_50_ or EC_50_), maximal response (E_max_), AUC, and Hill slope values, are summarized in Table 3.

### 3.6. Molecular Docking of Phenylaminojuglone ***AJ-D***

To obtain complementary mechanistic information regarding the spasmolytic activity of **AJ-D**, molecular docking simulations were performed using the L-type voltage-gated calcium channel Ca_V_1.2 (PDB ID: 8WE8), which is a molecular target directly involved in extracellular Ca^2+^ influx and smooth muscle contraction. Based on the functional pharmacological findings, verapamil was included as a reference L-type calcium channel blocker to provide a comparative pharmacological context.

As summarized in Table 4, **AJ-D** displayed a favorable predicted binding free energy within the selected Ca_V_1.2 transmembrane cavity (ΔE_bind_ = −7.0 kcal·mol^−1^), comparable to that of verapamil (ΔE_bind_ = −7.2 kcal·mol^−1^). Although docking energies should not be interpreted as direct measures of biological potency, these values suggest that **AJ-D** can be stably accommodated within the Ca_V_1.2 binding region.

The predicted binding conformations of **AJ-D** and verapamil revealed distinct hydrogen-bonding patterns while occupying a similar hydrophobic region within the Ca_V_1.2 transmembrane cavity. Both ligands shared hydrophobic contacts with residues Val1565, Leu1591, Met1596, Val1604, and Ala1612 (Figure S8, Supplementary Materials), suggesting a partial overlap within the predicted binding site. In contrast, differences in hydrogen bonding interactions were observed, with **AJ-D** interacting primarily with Asn1595 and Met1596, whereas verapamil interacted with Cys1588 and Arg1590.

**Figure 5:**
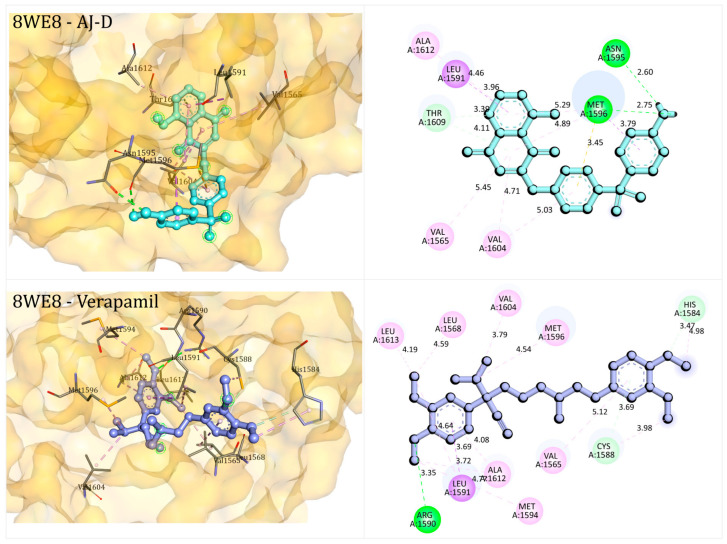
Predicted ligand–residue interactions of **AJ-D** and verapamil within the Ca_V_1.2 L-type voltage-gated calcium channel (PDB ID: 8WE8). Three-dimensional representations and the corresponding two-dimensional interaction maps are shown. Hydrogen atoms are omitted for clarity. Green: conventional hydrogen bonds; light green: carbon–hydrogen or π-donor hydrogen bonds; pink: alkyl/π–alkyl interactions; purple: π–σ interactions.

Figure 5 illustrates the predicted ligand–residue interactions of **AJ-D** and verapamil within the Ca_V_1.2 binding cavity. **AJ-D** established hydrogen-bonding interactions with Asn1595 and Met1596, along with hydrophobic contacts involving Val1565, Leu1591, Val1604, and Ala1612. Similarly, verapamil formed hydrogen-bond interactions with Arg1590 and Cys1588 and hydrophobic interactions with residues including Leu1568, Leu1613, Val1604, Met1596, and Ala1612. Overall, both ligands occupied overlapping regions of the Ca_V_1.2 cavity while exhibiting distinct interaction patterns with the residues.

### 3.7. DFT-Based Electronic Structure Analysis of ***AJ-D*** and Verapamil

The frontier molecular orbital (FMO) analysis of **AJ-D** and verapamil, computed at the M06-2X/6-311++G(d,p) level of theory under Koopmans’ approximation, revealed significant differences in their electronic structures (Figure S9, Supplementary Materials). **AJ-D** presented a HOMO energy of −7.48 eV and a LUMO energy of −2.26 eV, resulting in a HOMO–LUMO energy gap of 5.23 eV. In contrast, verapamil exhibited a HOMO energy of −6.82 eV and a LUMO energy of −0.32 eV, corresponding to a larger energy gap of 6.50 eV. These results indicate differences in the electronic structures of the two compounds.

Regarding orbital localization, the HOMO of **AJ-D** is broadly delocalized over the naphthoquinone framework, bridging the amino group, aromatic rings of the dapsone fragment, and sulfone functionality, indicating extensive π-electron conjugation throughout the molecule. In contrast, the LUMO was predominantly localized on the naphthoquinone core, particularly around the quinone carbonyl groups, identifying this region as the principal electron-accepting domain. For verapamil, both the HOMO and LUMO orbitals are mainly distributed over the methoxy-substituted aromatic rings, highlighting the dominant contribution of the aromatic π-system to its frontier orbital distribution.

The global reactivity indices calculated using Koopmans’ approximation are summarized in Table 5. **AJ-D** exhibited a higher ionization potential (*IP* = 7.48 eV) and electron affinity (*EA* = 2.26 eV) than verapamil (*IP* = 6.82 eV; *EA* = 0.32 eV), indicating a greater tendency to accept electronic density while requiring more energy for electron removal. In agreement with the HOMO–LUMO analysis, verapamil displayed a larger global hardness value (*η* = 3.25 eV) than **AJ-D** (*η* = 2.61 eV), reflecting a greater resistance to electronic perturbation.

Notably, the electrophilicity index of **AJ-D** (ω = 4.54 eV) is substantially higher than that of verapamil (ω = 1.96 eV), and the values of the electron-donating (ω^−^ = 7.31 eV), electron-accepting (ω^+^ = 2.44 eV), and net electrophilicity (Δω^±^ = 9.75 eV) descriptors are larger.

The local reactivity of **AJ-D** and verapamil was further investigated using Fukui functions (*f*^+^, *f*^−^, and *f*^0^) and molecular electrostatic potential (MEP) surface analysis (Figure S10, Supplementary Materials). For **AJ-D**, the *f*^+^ isosurface, associated with susceptibility toward nucleophilic attack, is mainly concentrated over the naphthoquinone ring and quinone carbonyl groups, identifying these regions as the principal electrophilic centers of the molecule. In contrast, the *f*^−^ function, which identifies electron-rich regions susceptible to electrophilic attack, is distributed throughout the molecular framework, with particularly strong contributions from the sulfone oxygen atoms, amino nitrogen atoms, and the conjugated aromatic system. The *f*^0^ function, associated with radical reactivity, is predominantly localized over the central aromatic region connecting the naphthoquinone and diphenyl sulfone fragments, suggesting that radical processes may preferentially involve this conjugated domain. For verapamil, the *f*^+^ isosurface was mainly localized over one methoxy-substituted aromatic ring, whereas the *f*^−^ distribution extended across both aromatic rings and the tertiary amino-containing side chain, reflecting the electron-rich character of these regions.

The MEP surfaces further support this observation. **AJ-D** displays a pronounced negative electrostatic potential around the quinone carbonyl oxygen atoms, sulfone oxygen atoms, and amino functionalities, whereas regions of positive potential are localized near the hydrogen-bond donor groups and less electron-rich aromatic regions. Verapamil exhibits a more homogeneous electrostatic distribution with less pronounced negative potential regions. Collectively, these findings indicate a higher degree of electronic differentiation and localized charge concentration in **AJ-D** compared with verapamil.

### 3.8. Predicted ADMET Profile of ***AJ-D***

The pharmacokinetic and toxicity profiles of **AJ-D** were predicted using the pkCSM online platform, and the results are summarized in Table 6. This compound satisfies Lipinski’s rule of five, supporting its predicted oral drug-likeness.

The predicted Caco-2 permeability value for **AJ-D** was 0.58 log Papp, indicating a favorable intestinal permeability. Consistent with this result, the predicted human intestinal absorption (HIA) exceeded 80%, suggesting efficient oral absorption. In contrast, the predicted skin permeability value was low (log Kp = −2.74), indicating limited transdermal absorption.

The predicted volume of distribution at steady state (VDss) was −0.11 log L/kg, suggesting a moderate systemic distribution. The predicted blood–brain barrier permeability (log BB) was −1.29, indicating limited central nervous system penetration. Similarly, the predicted central nervous system permeability (log PS) was negative, further supporting the low probability of CNS exposure.

Regarding metabolism, **AJ-D** was predicted not to inhibit CYP2D6, whereas it was predicted to act as a CYP3A4 inhibitor, suggesting the possibility of metabolic interactions with drugs metabolized through this pathway.

The predicted total clearance was 0.09 log mL/min/kg, indicating a relatively slow systemic elimination. Finally, the predicted acute oral toxicity value (rat LD_50_) was 2.11, suggesting no major acute toxicity alerts according to the pkCSM predictive model.

To complement the ADMET analysis, an extended in silico toxicological assessment of **AJ-D** was performed using the ProTox-3.0 platform (Table 7). **AJ-D** exhibited a predicted oral median lethal dose (LD_50_) of 2000 mg/kg, corresponding to Toxicity Class 4, with a prediction accuracy of 67.38% and an average similarity of 55.15% relative to compounds within the training set. According to the ProTox classification system, this result suggests a moderately predicted acute oral toxicity profile, indicating that **AJ-D** does not fall within the highly toxic categories while still requiring further toxicological evaluation. Regarding specific toxicity endpoints, **AJ-D** was predicted to be inactive for mutagenicity, carcinogenicity, cytotoxicity, neurotoxicity, nephrotoxicity, cardiotoxicity, and respiratory toxicity. However, the compound was predicted to be active for immunotoxicity, with a relatively high probability score (0.81), and showed a borderline active prediction for hepatotoxicity (0.50). These alerts indicate that although **AJ-D** does not show major predicted genotoxic or cytotoxic liabilities, potential immunotoxic and hepatic risks should be considered in future experimental safety evaluations.

## 4. Discussion

The solvent-free mechanochemical amination of juglone with dapsone afforded **AJ-D** with complete regioselectivity for C-3 substitution. Compared with ethanol-based conditions, mechanical activation using a mini-rotary tube shaker reduced the reaction time by half while providing a comparable yield, highlighting the operational and kinetic advantages of solvent-free solid-state processes. The moderate yields obtained by both methods reflect the inherent selectivity of the reaction, which leads to the formation of only the desired regioisomer. The purification process used to isolate this product with high purity contributed to the final yield. Consistent with our previous reports on silica gel-mediated aza-Michael additions for the synthesis of phenylaminojuglones and structurally related phenylaminonaphthoquinones [17,18,53], the results further demonstrate the versatility of this mechanochemical strategy for the functionalization of the naphthoquinone scaffold. Mechanistically, silica gel likely acts as a mild acid [54], increasing the electrophilicity of the conjugated quinone system and facilitating nucleophilic attack at the β-carbon (C-3), thereby accounting for the observed regioselectivity.

From a pharmacological perspective, **AJ-D** exhibited significant relaxant effects on the isolated rat ileum under basal conditions and in tissues precontracted with ACh and KCl. Relaxation of ACh-induced contractions (pharmacomechanical coupling) and KCl-induced contractions (electromechanical coupling) suggests a mechanism operating downstream of receptor activation, which is compatible with the modulation of Ca^2+^ influx pathways [55,56,57]. The summarized pharmacological parameters further showed that **AJ-D** reduced contractile efficacy (E_max_) and produced rightward shifts in the concentration–response curves, particularly under ACh- and KCl-induced contraction conditions. These findings are consistent with interference in calcium-dependent contractile mechanisms rather than simple receptor antagonism [58].

Mechanistic experiments supported this hypothesis. The absence of significant effects following atropine pretreatment indicates that muscarinic receptor antagonism is unlikely to be the primary mechanism underlying **AJ-D**-induced relaxation. Similarly, the absence of significant changes following pretreatment with K^+^ channel blockers (BaCl_2_, glibenclamide, 4-AP, and TEA) suggests that membrane hyperpolarization through these channels does not substantially contribute to the observed relaxant response [59].

Conversely, verapamil significantly reduced **AJ-D**-mediated relaxation, and **AJ-D** markedly attenuated CaCl_2_-induced contractions under Ca^2+^-free/high-K^+^ conditions. These findings suggest that **AJ-D** interferes with extracellular calcium influx pathways and may involve voltage-dependent calcium entry mechanisms that regulate gastrointestinal smooth muscle contraction [60,61].

Despite incorporating the dapsone moiety into the phenylaminojuglone scaffold, **AJ-D** appears to preserve the pharmacological properties previously reported for juglone derivatives. Previous studies in vascular and intestinal smooth muscle preparations [17,62] demonstrated that juglone shifts CaCl_2_-induced contraction curves to the right under Ca^2+^-free conditions, a pharmacological profile consistent with reduced extracellular Ca^2+^ influx. Similar behavior has been reported for structurally related phenylaminojuglones [18], suggesting that the modulation of calcium-dependent contractility may represent a conserved pharmacological characteristic within this class of compounds.

From a structure–activity relationship (SAR) perspective, **AJ-D** represents a strategic structural expansion of the phenylaminojuglone scaffold by incorporating a dapsone-derived diphenyl sulfone moiety. A comparison with structurally related phenylaminojuglone derivatives previously evaluated by our research group under identical experimental conditions in isolated rat ileum revealed distinct pharmacological profiles across this chemical series.

While the parent compound juglone displays a dual mechanism involving muscarinic receptor antagonism and calcium channel blockade [17], the unsubstituted phenylaminojuglone derivative **AJ-2** induces smooth muscle relaxation through activation of β-adrenergic signaling, the NO–sGC–cGMP pathway, and K^+^ channel opening [18]. In contrast, bulkier C-3 substituents, such as the 4-methoxyphenyl (**AJ-8**) and 3,4,5-trimethoxyphenyl (**AJ-11**) groups, markedly reduced spasmolytic potency, highlighting the importance of steric and electronic factors in modulating the biological activity. The incorporation of a dapsone-derived diphenyl sulfone moiety into **AJ-D** resulted in a distinct pharmacodynamic profile while preserving potent spasmolytic activity.

Despite its substantially larger size and greater steric bulk relative to the phenyl group of **AJ-2**, **AJ-D** retained potent spasmolytic and antispasmodic activities, supporting the 1,4-naphthoquinone core and C-3 arylamino linkage as key pharmacophoric elements for smooth muscle relaxation. Unlike juglone, **AJ-D** showed no evidence of muscarinic receptor antagonism, as atropine pretreatment did not significantly modify its relaxant effect. Similarly, co-administration of K^+^ channel blockers failed to significantly alter **AJ-D**-induced relaxation, suggesting that K^+^ channel activation, a major mechanism underlying the activity of **AJ-2** [18], does not substantially contribute to the pharmacological effects of **AJ-D**. Collectively, these findings indicate that the antispasmodic activity of **AJ-D** is predominantly associated with the inhibition of extracellular Ca^2+^ influx through voltage-dependent calcium channels.

The β-adrenergic and nitrergic (NO–sGC–cGMP) pathways, which contribute significantly to the spasmolytic activity of **AJ-2**, were not evaluated for **AJ-D** in the present study and, therefore, cannot be excluded. Nevertheless, the functional pharmacology and molecular docking results support the hypothesis that incorporation of the diphenyl sulfone moiety modifies the steric and electronic properties of the phenylaminojuglone scaffold, favoring interactions within the Ca_V_1.2 binding cavity and shifting the pharmacological profile from the broader, multitarget behavior observed in other phenylaminojuglone derivatives (e.g., **AJ-2**, **AJ-8**, and **AJ-11**) toward a mechanism in which inhibition of extracellular Ca^2+^ influx through voltage-dependent calcium channels appears to predominate.

Molecular docking simulations provided complementary structural information supporting the functional and pharmacological findings. **AJ-D** exhibited a favorable predicted binding free energy within the selected Ca_V_1.2 cavity (ΔE_bind_ = −7.0 kcal·mol^−1^), comparable to that of the reference calcium channel blocker verapamil (Δ_Ebind_ = −7.2 kcal·mol^−1^). Although these docking energies cannot be directly interpreted as measures of pharmacological potency [63,64], they suggest that **AJ-D** can stably accommodate within the Ca_V_1.2 binding region. Interestingly, both ligands shared hydrophobic interactions with residues Val1565, Leu1591, Met1596, Val1604, and Ala1612, indicating a partial overlap within the predicted binding cavity. In contrast, distinct hydrogen-bonding patterns were observed, with **AJ-D** interacting primarily with Asn1595 and Met1596, whereas verapamil interacted with Cys1588 and Arg1590. The overlap in hydrophobic interactions, together with differences in specific polar contacts, suggests that **AJ-D** and verapamil occupy similar regions of the Ca_V_1.2 cavity while exhibiting distinct interaction profiles. These computational findings are consistent with functional experiments showing that verapamil reduced **AJ-D** potency and that **AJ-D** attenuated Ca^2+^-dependent contractile responses.

Interestingly, despite exhibiting a docking score similar to that of verapamil, **AJ-D** displayed lower global hardness, higher electrophilicity, and a smaller HOMO–LUMO energy gap according to conceptual DFT descriptors. The classification of **AJ-D** as a strong electrophile under the Domingo scale [65] suggests an enhanced ability to participate in charge-transfer interactions and electronic polarization processes. Consistent with this behavior, the electron-donating, electron-accepting, and net electrophilicity descriptors collectively indicate an increased capacity of **AJ-D** to accept and redistribute electronic density within its molecular environment. Furthermore, the reduced HOMO–LUMO gap reflects greater electronic polarizability and a higher propensity for intermolecular interactions. The MEP surface revealed pronounced negative electrostatic potential regions localized around the quinone carbonyls and sulfone oxygen atoms, identifying these functionalities as potential interaction hotspots. Collectively, these electronic features provide a coherent quantum chemical framework that complements the pharmacological and docking results and may contribute to ligand recognition and stabilization within the predicted Ca_V_1.2 binding cavity.

Nevertheless, the convergence of pharmacological experiments, molecular docking simulations, and DFT analyses suggests that **AJ-D** interferes with extracellular calcium influx pathways and may involve voltage-dependent calcium entry mechanisms, contributing to its relaxant activity.

In silico ADMET and ProTox predictions suggested that **AJ-D** satisfies Lipinski’s rule of five [66] and exhibits favorable intestinal absorption and limited BBB penetration [67], supporting its potential as a peripheral gastrointestinal relaxant. However, the predicted CYP3A4 inhibition [68,69], along with the immunotoxicity signal and borderline hepatotoxicity alert, warrants further experimental investigation. These predictions should, however, be interpreted cautiously until confirmed through experimental pharmacokinetic and toxicological studies.

The present study has several limitations that should be acknowledged. First, a direct pharmacological comparison based on full concentration–response curves for verapamil was not performed; therefore, the quantitative potency comparisons should be interpreted cautiously. Second, the molecular docking and DFT analyses provide valuable mechanistic insights but should be regarded as hypothesis-generating approaches rather than direct evidence of target engagement. Confirmation of Ca_V_1.2 modulation requires dedicated electrophysiological studies and target-validation assays.

Another limitation is that intracellular Ca^2+^ release pathways, including IP_3_ receptor- and ryanodine receptor-sensitive stores, were not directly investigated. This experimental strategy was guided by our previous findings with juglone, the parent compound of **AJ-D**, in which Ca^2+^-free experiments demonstrated that intracellular Ca^2+^ mobilization was not significantly affected, whereas inhibition of extracellular Ca^2+^ influx was identified as the predominant mechanism underlying its spasmolytic activity [17]. Based on these observations, **AJ-D** was designed as a structural derivative of juglone to determine whether this predominant mechanism was retained after incorporating the dapsone-derived moiety. Although the present findings support a major contribution of extracellular Ca^2+^ influx inhibition, the involvement of intracellular Ca^2+^ release mechanisms cannot be ruled out. Therefore, future studies using selective modulators of IP_3_ and ryanodine receptors are required to establish the contribution of intracellular Ca^2+^ stores to the spasmolytic activity of **AJ-D**.

Finally, pharmacological evaluation was restricted to an isolated rat ileum model and was not supported by in vivo efficacy, pharmacokinetic, or toxicological studies. Consequently, the translational and therapeutic relevance of the present findings remains to be established through additional preclinical studies.

In general, the incorporation of the dapsone moiety into the juglone scaffold generated a derivative exhibiting significant spasmolytic activity and a potentially favorable predicted pharmacokinetic profile. Although additional experimental validation is required, our findings support **AJ-D** as a relevant scaffold for the further development of smooth muscle relaxants.

## 5. Conclusions

**AJ-D** was successfully synthesized through a solvent-free mechanochemical aza-Michael reaction using silica gel as a solid support, affording a single C-3 regioisomer with shorter reaction times compared to those under ethanol-based conditions. Pharmacological evaluation demonstrated that **AJ-D** exhibited concentration-dependent spasmolytic and antispasmodic activities in isolated rat ileum preparations. Mechanistic studies indicated that muscarinic receptors and major K^+^ channel subtypes do not play a major role in the observed relaxation. In contrast, functional experiments involving verapamil and Ca^2+^ reintroduction protocols suggested that the activity of **AJ-D** is compatible with the modulation of extracellular calcium influx pathways involved in smooth muscle contraction. However, direct confirmation of the molecular targets involved will require dedicated electrophysiological and mechanistic studies.

The integration of pharmacological experiments, molecular docking, ADMET prediction, and DFT calculations consistently supports the role of **AJ-D** as a promising antispasmodic scaffold. The convergence of pharmacological and computational evidence suggests that **AJ-D** interferes with extracellular calcium influx pathways and may involve voltage-dependent calcium entry mechanisms, potentially including Ca_V_1.2 channels.

## Figures and Tables

**Table 1:** Summary of equations used to calculate various global reactivity indexes in the TAFF pipeline [44]. These indices were calculated using CDFT descriptors derived from the HOMO ($\epsilon_{H}$) and LUMO ($\epsilon_{L}$) energies.

Global Reactivity Descriptors	Koopmans’ Theorem	Reference
Ionization potential (*IP*)	$I=-\epsilon_{H}$	[45,46]
Electron affinity (*EA*)	$A=-\epsilon_{L}$	[45,46]
Global Hardness (*η*)	$\eta =\frac{1}{2}\left(\epsilon_{L}-\epsilon_{H}\right)$	[45,46]
Electronegativity (*χ*)	$\chi =-\frac{1}{2}\left(\epsilon_{L}+\epsilon_{H}\right)$	[47,48,49]
Electrophilicity (*ω*)	$\omega =\frac{\mu^{2}}{2\eta }=\frac{{\left(\epsilon_{L}+\epsilon_{H}\right)}^{2}}{2(\epsilon_{L}-\epsilon_{H})}$	[50]
Electron Acceptor (*ω*^+^)	$\omega^{+}=\frac{{\left(\epsilon_{L}+{3\epsilon }_{H}\right)}^{2}}{16(\epsilon_{L}-\epsilon_{H})}$	[50]
Electron Donator (*ω*^−^)	$\omega^{-}=\frac{{\left({3\epsilon }_{L}+\epsilon_{H}\right)}^{2}}{16(\epsilon_{L}-\epsilon_{H})}$	[50]
Net Electrophilicity (Δ*ω*^±^)	${∆\omega }^{\pm }=\omega^{+}+\omega^{-}$	[51]

**Table 2:** Comparison of reaction yields and reaction times for phenylaminojuglone **AJ-D** synthesized under solvent-free and solvent-based conditions.

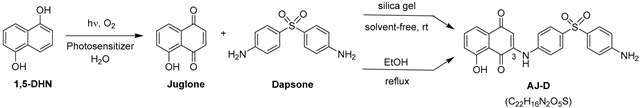				
Compound	Solvent-Free		Solvent (EtOH)	
	Yield (%)	Time (h)	Yield (%)	Time (h)
**AJ-D**	51.3	3.0	53.0	6.0

**Table 3:** Pharmacological parameters of **AJ-D** in isolated rat ileal preparations.

Experimental Condition	Parameter	Control	AJ-D (10^−5^ M)	AJ-D (10^−4^ M)
ACh-induced contractions	pEC_50_	6.83 ± 0.10	6.72 ± 0.13	6.23 ± 0.16 **
	E_max_ (%)	119.5 ± 3.8	127.9 ± 5.1	82.4 ± 4.8 ***
	AUC (a.u.)	348 ± 24	403 ± 41	193 ± 25 **
	EC_50_ (M)	1.49 × 10^−7^	1.89 × 10^−7^	5.86 × 10^−7^ **
KCl-induced contractions	EC_50_ (mM)	14.2 ± 1.0	16.2 ± 1.3	48.4 ± 3.3 ***
	E_max_ (%)	80.9 ± 4.9	84.6 ± 4.5	55.4 ± 4.9 **
	AUC (a.u.)	299 ± 17	299 ± 20	153 ± 15 ***
	Hill slope	1.57 ± 0.17	1.59 ± 0.18	1.31 ± 0.12
CaCl_2_-induced contractions	EC_50_ (mM)	2.67 ± 0.20	2.32 ± 0.15	145 ± 72 *
	E_max_ (%)	63.8 ± 3.5	64.6 ± 2.9	20.1 ± 2.0 ***
	AUC (a.u.)	158 ± 10	167 ± 10	59 ± 5 ***
	Hill slope	0.75 ± 0.05	0.74 ± 0.04	0.41 ± 0.04 *

* No statistically significant difference compared to the control group. ** *p* < 0.01 versus control. *** *p* < 0.001 versus control. AUC: area under the concentration–response curve.

**Table 4:** Predicted binding free energies (ΔE_bind_, kcal·mol^−1^) and main ligand–residue interactions obtained from molecular docking calculations of **AJ-D** and verapamil against the Ca_V_1.2 L-type voltage-gated calcium channel (PDB ID: 8WE8).

Ligand	ΔE_Bind_ (kcal.mol^−1^)	Hydrogen-Bond Residues	Main Hydrophobic Contact Residues
**AJ-D**	−7.0	Asn1595, Met1596	Val 1565, Leu 1591, Met 1596, Val 1604, Ala 1612
Verapamil	−7.2	Cys1588, Arg1590	

**Table 5:** The global reactivity indices (in eV) for **AJ-D** and verapamil were obtained using Koopman’s approximation.

Molecules	$\epsilon_{H}$	$\epsilon_{L}$	GAP	*IP*	*EA*	*η*	*ω*	*χ*	*ω* ^+^	*ω* ^−^	Δ*ω*^±^
**AJ-D**	−7.48	−2.26	5.23	7.48	2.26	2.61	4.54	4.87	2.44	7.31	9.75
Verapamil	−6.82	−0.32	6.50	6.82	0.32	3.25	1.96	3.57	0.58	4.15	4.73

**Table 6:** ADMET properties of compound **AJ-D**.

	Property									
	Absorption			Distribution			Metabolism	Excretion	Toxicity	
	Model Name									
Compound	Caco-2	IA	SP	VD ss	BBB	CNS	CYP2D6/ CYP3A4 Inhibitor	TC	Oral Rat Acute Tox. (LD_50_)	Oral Rat Chronic Tox. -LOAEL
**AJ-D**	0.58	82.46	−2.74	−0.11	−1.29	−2.28	No/Yes	0.09	2.11	2.17

**Caco-2**: Caucasian colon adenocarcinoma permeability (Log Papp in 10^−6^ cm/s); **IA**: Intestinal Absorption (% Absorbed); **SP**: Skin Permeability (log Kp); **VDss**: steady-state Volume of Distribution (Log L/kg); **BBB**: Blood–Brain Barrier permeability (Log BB); CNS: Central Nervous System (Log PS); **CYP2D6**: Cytochrome P450 2D6 inhibitor; **CYP3A4**: Cytochrome P450 3A4 inhibitor; **TC**: Total Clearance (Log mL/min/kg); **LD_50_**: Lethal Dose, 50% (mol/Kg); **LOAEL**: Lowest Observed Adverse Effect Level (Log mg/kg_bw/day).

**Table 7:** Extended in silico toxicity profile of **AJ-D** predicted using ProTox-3.0.

Toxicity Classification	Target Endpoint	Prediction	Probability/Value
Acute Toxicity	Predicted Oral Rat LD_50_	–	2000 mg/kg
	Predicted Toxicity Class	–	Class 4
	Prediction Accuracy	–	67.38%
	Average Similarity	–	55.15%
Organ Toxicity	Hepatotoxicity (DILI)	Active	0.50
	Neurotoxicity	Inactive	0.89
	Nephrotoxicity	Inactive	0.53
	Cardiotoxicity	Inactive	0.73
	Respiratory toxicity	Inactive	0.63
Toxicity Endpoints	Carcinogenicity	Inactive	0.60
	Immunotoxicity	Active	0.81
	Mutagenicity (Ames test)	Inactive	0.55
	Cytotoxicity	Inactive	0.71

DILI = Drug-Induced Liver Injury. – = Not Applicable.

## Data Availability

Data are contained within the article and Supplementary Materials.

## Supplementary Materials

The following supporting information can be downloaded at: https://www.mdpi.com/article/10.3390/biom16071045/s1, Figure S1: IR spectrum of **AJ-D**; Figure S2: 1H NMR (400 MHz, DMSO-d6) spectrum of **AJ-D**; Figure S3: ^13^C NMR (100 MHz, DMSO-d6) spectrum of **AJ-D**; Figure S4: HMBC spectrum of **AJ-D**; Figure S5: HRMS (APCI) full-scan spectrum of **AJ-D**; Figure S6: Muscarinic receptor involvement in the relaxant effect of **AJ-D** on rat ileum; Figure S7: Effects of K^+^ channel blockers on **AJ-D**-induced relaxation in rat ileum; Figure S8: Predicted binding conformations of **AJ-D** and verapamil within the CaV1.2 L-type calcium channel; Figure S9: Frontier molecular orbitals and HOMO–LUMO energy gaps of **AJ-D** and verapamil; Figure S10: Molecular electrostatic potential maps and Fukui functions of **AJ-D** and verapamil.

## Abbreviations

The following abbreviations are used in this manuscript:

ACh	Acetylcholine
ADMET	Absorption, Distribution, Metabolism, Excretion, and Toxicity
AUC	Area Under the Curve
DFT	Density Functional Theory
EC_50_	Half-maximal Effective Concentration
HOMO	Highest Occupied Molecular Orbital
LD_50_	Median Lethal Dose
LUMO	Lowest Unoccupied Molecular Orbital
MEP	Molecular Electrostatic Potential
PDB	Protein Data Bank
pEC_50_	The negative logarithm of the EC_50_
RMSD	Root Mean Square Deviation
SAR	Structure–Activity Relationship
