# Supplementary material for: Solvent-Free Mechanochemical Synthesis, Antispasmodic Activity, and Integrated In Silico Mechanistic Analysis of a Dapsone-Derived Phenylaminojuglone
Source: Biomolecules. 2026 Jul 17;16(7):1045. doi: 10.3390/biom16071045 (PMC13406440; doi:10.3390/biom16071045)
Supplement: Supplementary file 1 [file biomolecules-16-01045-s001.zip › biomolecules-4400423-supplementary.pdf]

Solvent-Free Mechanochemical Synthesis, Antispasmodic Activity, and Integrated In Silico Mechanistic Analysis of a Dapsone-Derived Phenylaminojuglone

Ricardo E. Zavaleta-Miñano [1], Elena Mantilla-Rodríguez [1], Roberto O. Ybañez-Julca [1,*], Daniel Asunción-Alvarez [1], Cinthya Enriquez-Lara [2], Justo Huertas-Córdova [2], Iván M. Quispe-Díaz [1], Rafael Jara-Aguilar [1], Edison Vásquez-Corales [3], Wilfredo O. Gutiérrez-Alvarado [4], Osvaldo Yañez [5], and Julio Benites [2,6,*]

1 Grupo de Investigación en Estudios de Compuestos Naturales y Sintéticos con Actividad a Nivel Sistema Nervioso Central y Musculo Liso, Laboratorio de Farmacología, Facultad de Farmacia y Bioquímica, Universidad Nacional de Trujillo, Trujillo 13011, Perú; rezavaletami@unitru.edu.pe (R.E.Z.-M.); amantilla@unitru.edu.pe (E.M-R.); rybanez@unitru.edu.pe (R.O.Y.-J.); hasuncion@unitru.edu.pe (D.A.-A.); iquispe@unitru.edu.pe (I.M.Q.-D.); djara@unitru.edu.pe (R.J.-A.).

2 Programa de Doctorado en Química Medicinal, Facultad de Ciencias de la Salud, Universidad Arturo Prat, Casilla 121, Iquique 1110939, Chile; cenriquez@estudiantesunap.cl (C.E.-L.); jhuertas@estudiantesunap.cl (J.H.-C.).

3 Vicerrectorado de Investigación y Postgrado, Universidad Católica Los Ángeles de Chimbote, Chimbote 02801, Perú; evasquezc@uladech.edu.pe (E.V.-C.).

4 Facultad de Farmacia y Bioquímica, Universidad Nacional de la Amazonía Peruana, Iquitos 16001, Perú; wilfredo.gutierrez@unapiquitos.edu.pe (W.O.G.-A).

5 Centro de Modelación Ambiental y Dinámica de Sistemas (CEMADIS), Facultad de Ingeniería y Negocios, Universidad de Las Américas, Santiago 7500975, Chile; oyanez@udla.cl (O.Y.).

6 Laboratorio de Química Medicinal, Química y Farmacia, Facultad de Ciencias de la Salud, Universidad Arturo Prat, Casilla 121, Iquique 1110939, Chile; juliob@unap.cl (J.B.).

* Correspondence: rybanez@unitru.edu.pe (R.O.Y.-J.); juliob@unap.cl (J.B.); Tel.: +51-976-345-993 (R.O.Y.-J.); +56-9-98277790 (J.B.)

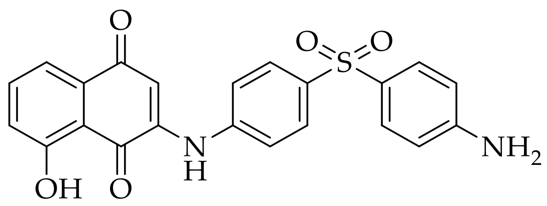

AJ-D

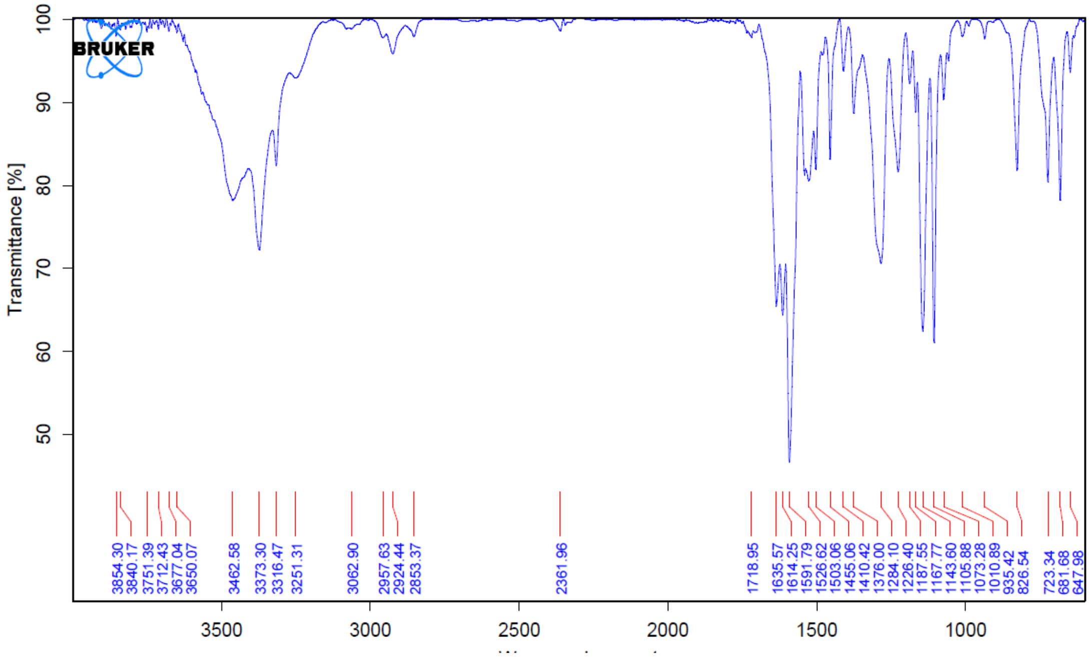

Figure S1. IR spectrum of **AJ-D**.

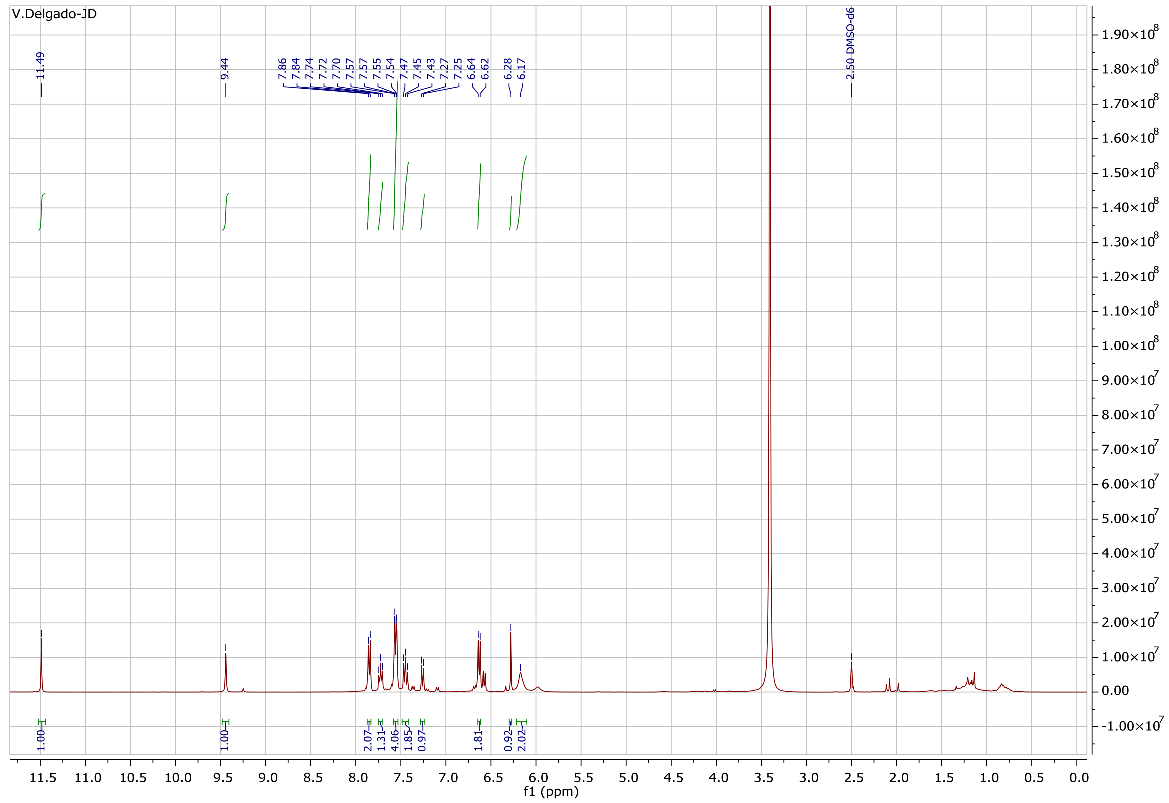

Figure S2. ^{1}H NMR (400 MHz, DMSO-d_6) spectrum of **AJ-D**.

* Note: The minor signals observed in the region of δ_H 0.6–2.1 ppm correspond to residual silicone grease/trace hydrocarbon contaminants from laboratory equipment and do not belong to compound **AJ-D** or synthesis by-products.

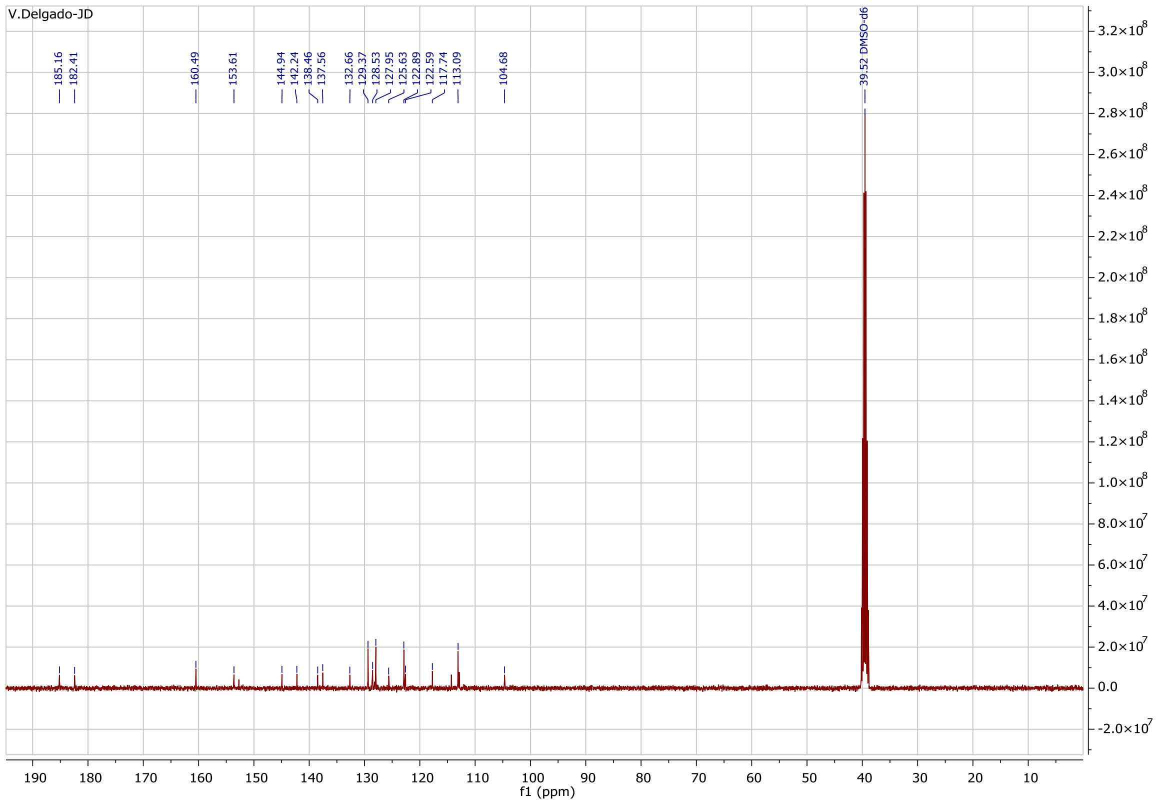

Figure S3. ^{13}C NMR (100 MHz, DMSO-d_6) spectrum of **AJ-D**.

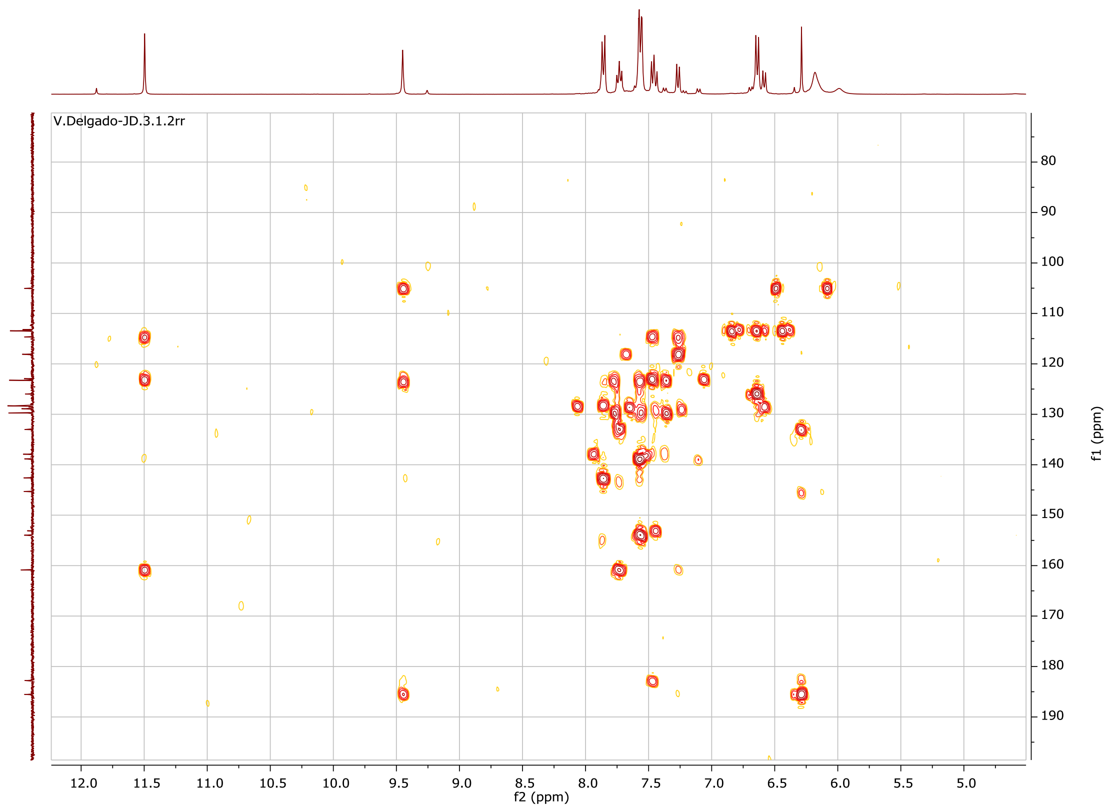

Figure S4. HMBC spectrum (400 MHz, DMSO-d_6) of **AJ-D**.

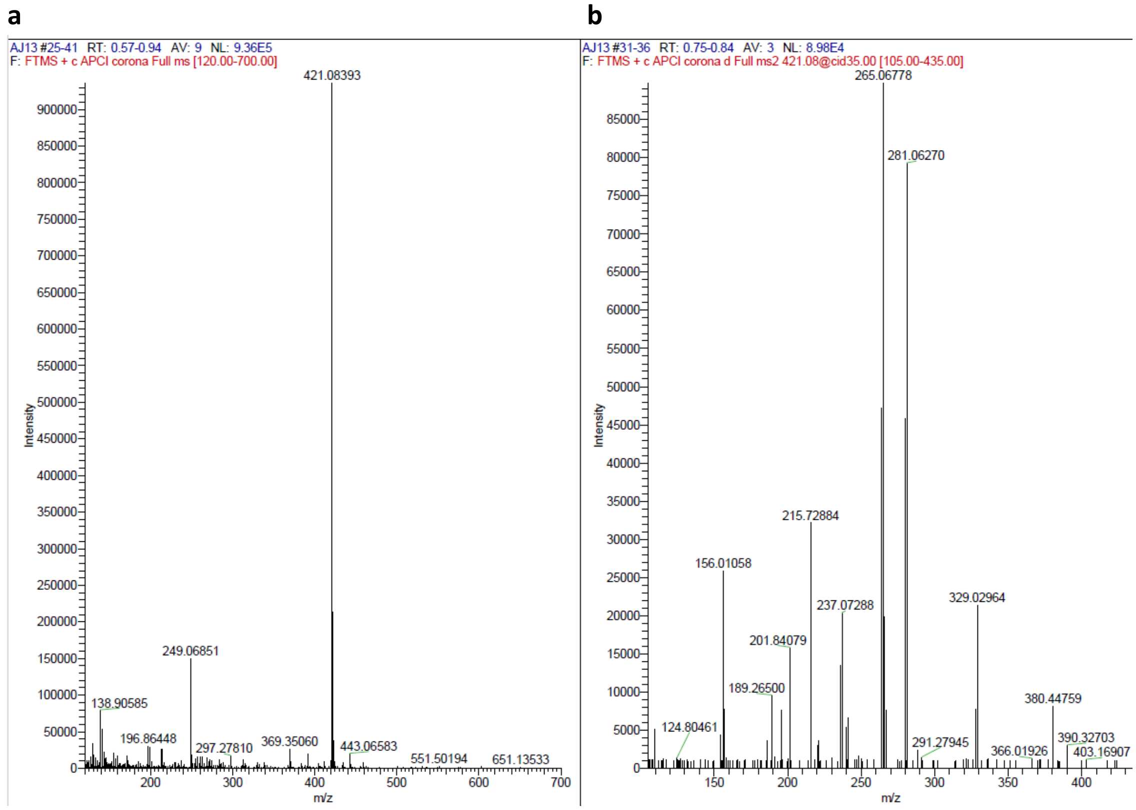

Figure S5a. HRMS (APCI) full-scan spectrum of **AJ-D. Figure S5b**: MS/MS fragmentation spectrum of AJ-D.

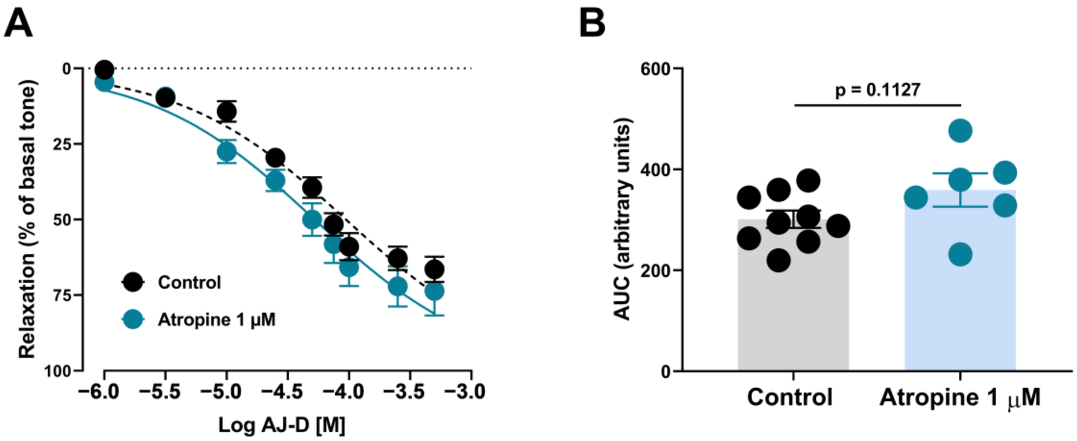

Figure S6. Involvement of muscarinic receptors in the relaxant effect of phenylaminojuglone **AJ-D** on rat ileum. (**A**) Concentration–response curves showing the relaxant effect of **AJ-D** on basal ileal tone under control conditions and after pre-incubation with atropine (1 μM). (**B**) Area under the concentration–response curve (AUC) used to quantify the overall relaxant response to **AJ-D** under control conditions and after atropine pretreatment. Data are expressed as mean ± SEM of the percentage of relaxation relative to the basal tone (n = 6–9 ileal segments). The colored lines represent the nonlinear regression fits using the Hill equation. Dose–response curves were analyzed using two-way ANOVA followed by Bonferroni's *post hoc* test, whereas AUC values were compared using an unpaired Student's t-test.

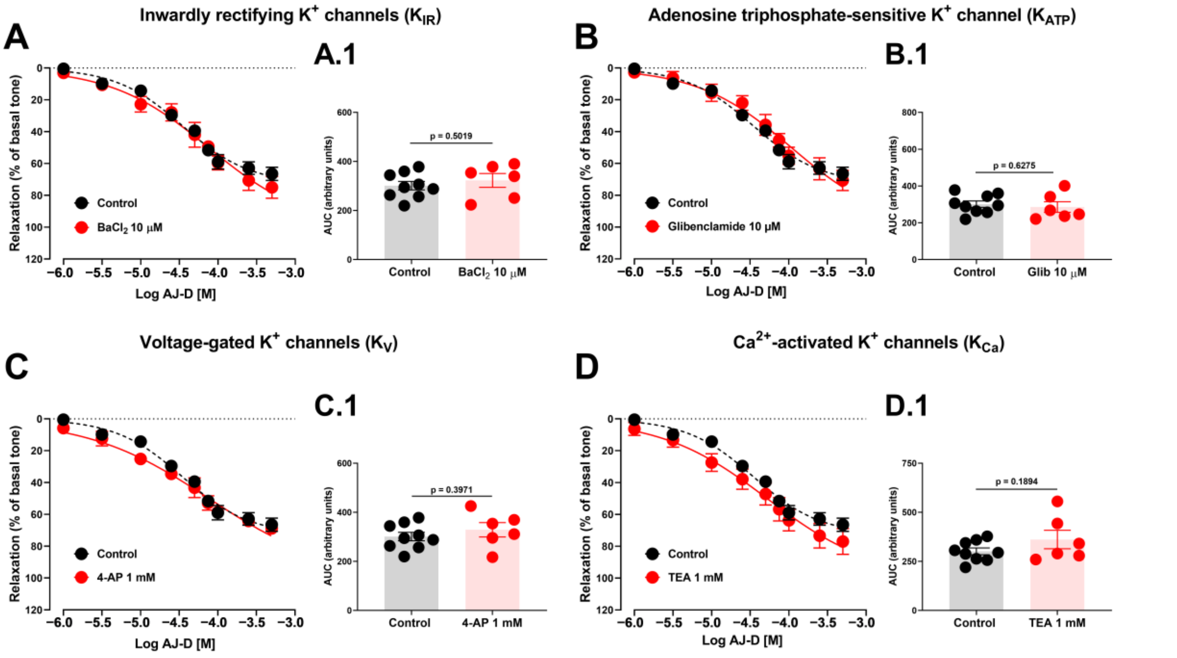

Figure S7. Effects of K^+ channel blockers on **AJ-D**–induced relaxation of rat ileal smooth muscle. (**A**) Concentration–response curves showing relaxation responses (% relaxation relative to basal tone) in the absence (control) and after preincubation with (**A**) $BaCl_2$ (10 μM), (**B**) glibenclamide (10 μM), (**C**) 4-aminopyridine (4-AP, 1 mM), and (**D**) tetraethylammonium (TEA, 1 mM) in the rat ileum. The corresponding area under the concentration–response curves (AUC) for control and blocker-pretreated tissues are shown on the right side of each panel (**A.1–D.1**). Each point in the concentration–response curves represents the mean± SEM of the contractile response (n = 6–9 ileal segments). Color lines represent nonlinear regression fits using the Hill equation. Dose–response curves were analyzed using two-way ANOVA followed by Bonferroni's *post hoc* test, and the AUC values were compared using an unpaired Student's t-test.

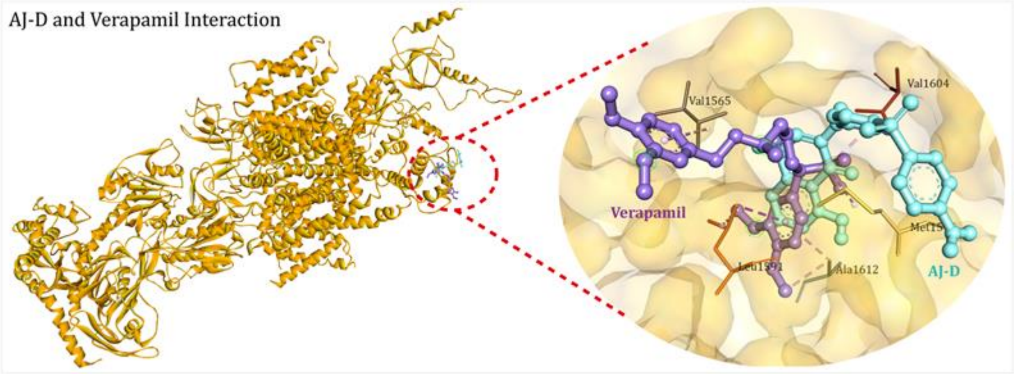

Figure S8. Predicted three-dimensional binding conformations of **AJ-D** and verapamil within the transmembrane cavity of the Cav1.2 L-type voltage-gated calcium channel (PDB ID: 8WE8). The figure illustrates the relative accommodation of both ligands within the selected binding region used in the docking analysis.

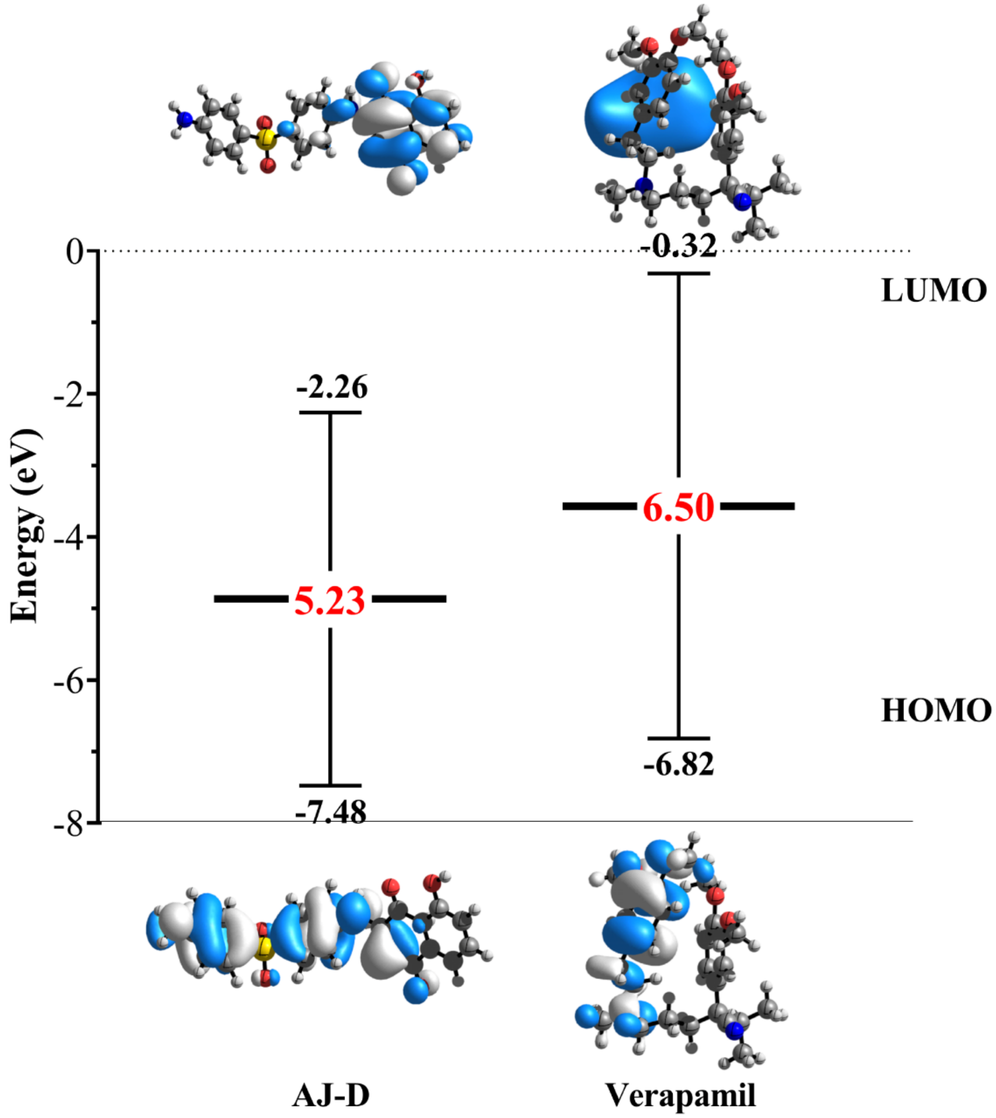

Figure S9. Isosurface representations of the frontier molecular orbitals and HOMO–LUMO energy gaps of **AJ-D** and verapamil.

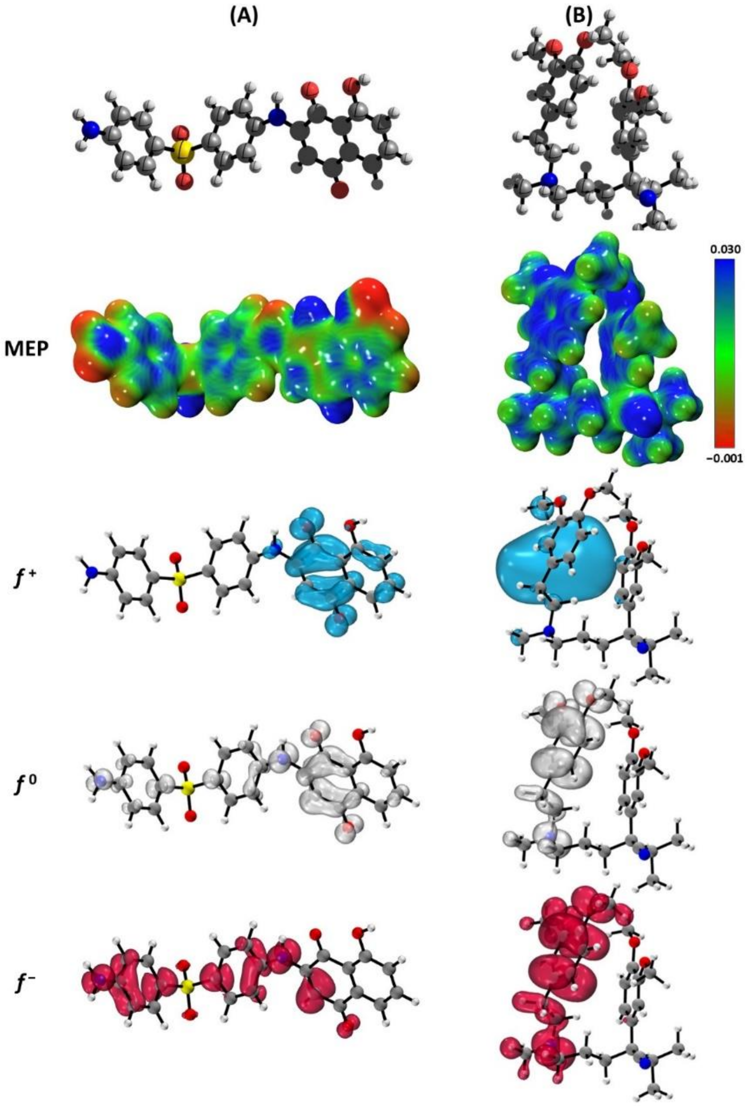

Figure S10. Isosurface representation of the molecular electrostatic potential maps and electrophilic f^-, radical f^0, and nucleophilic f^+ Fukui functions, for (**A**) AJ-D and (**B**) verapamil. The electrostatic potentials were mapped on the surface of the electron density of 0.04 a.u. The red surface corresponds to the negative region of the

electrostatic potential (−0.001 a.u.), whereas the blue surface corresponds to the region where the potential is positive (0.03 a.u.).
